# Characterization of Various Titanium-Dioxide-Based Catalysts Regarding Photocatalytic Mineralization of Carbamazepine also Combined with Ozonation

**DOI:** 10.3390/molecules27228041

**Published:** 2022-11-19

**Authors:** Gábor Kocsis, Erzsébet Szabó-Bárdos, Orsolya Fónagy, Evelin Farsang, Tatjána Juzsakova, Miklós Jakab, Péter Pekker, Margit Kovács, Ottó Horváth

**Affiliations:** 1 Environmental and Inorganic Photochemistry Research Group, Center for Natural Sciences, University of Pannonia, P.O. Box 1158, H-8210 Veszprém, Hungary; 2 Analytical Chemistry Research Group, Center for Natural Sciences, University of Pannonia, P.O. Box 1158, H-8210 Veszprém, Hungary; 3 Sustainability Solutions Research Lab, Research Center for Biochemical, Environmental and Chemical Engineering, University of Pannonia, P.O. Box 1158, H-8210 Veszprém, Hungary; 4 Department of Materials Engineering, Research Center for Engineering Sciences, University of Pannonia, P.O. Box 1158, H-8210 Veszprém, Hungary; 5 Environmental Mineralogy Research Group, Research Institute of Biomolecular and Chemical Engineering, University of Pannonia, P.O. Box 1158, H-8210 Veszprém, Hungary

**Keywords:** silver/nitrogen-doped TiO_2_, UV-vis-light-driven photocatalysis, carbamazepine degradation, ozonation, synergism, toxicity

## Abstract

Titanium-dioxide-based semiconductors proved to be appropriate for photocatalytic application to efficiently degrade emerging organic pollutants such as various herbicides, pesticides, and pharmaceuticals in waters of environmental importance. The characterization of various TiO_2_ catalysts, both bare and modified (Ag- and/or N-doped), by mechanochemical treatment was carried out in this work, regarding their structure, morphology, and photocatalytic activity. For the latter investigations, carbamazepine, an antidepressant, proved to be applicable and versatile. The photocatalytic behavior of the catalysts was studied under both UV and visible light. Besides the decomposition efficiency, monitoring the intermediates provided information on the degradation mechanisms. Mechanochemical treatment significantly increased the particle size (from 30 nm to 10 μm), causing a considerable (0.14 eV) decrease in the band gap. Depending on the irradiation wavelength and the catalyst, the activity orders differed, indicating that, in the mineralization processes of carbamazepine, the importance of the different oxidizing radicals considerably deviated, e.g., Ag-TiO_2_ < DP25-TiO_2_ < ground-DP25-TiO_2_ < N-TiO_2_ ≈ N-Ag-TiO_2_ for O_2_^•−^ and N-TiO_2_ ≈ Ag-TiO_2_ < N-Ag-TiO_2_ < ground-DP25-TiO_2_ ≈ DP25-TiO_2_ for HO^•^ generation under UV irradiation. Toxicity studies have shown that the resulting intermediates are more toxic than the starting drug molecule, so full mineralization is required. This could be realized by a synergistic combination of heterogeneous photocatalysis and ozonation.

## 1. Introduction

Many organic contaminants such as pesticides, pharmaceuticals, personal care products, and their metabolites are continuously released into the environment from various sources. In order to reduce human and environmental risks, the concentration of these micropollutants should be minimized, especially in the effluents from wastewater treatment plants. For the improvement in their removal, conventional technologies can be combined with modern purification methods, e.g., advanced oxidation processes (AOPs), which generate oxidative free radicals, mostly reactive oxygen species (ROS). Heterogeneous photocatalytic AOPs are the most studied in the field of the removal of pharmaceutical residues from water [1]. They can also be powered by solar energy, which not only reduces the cost but also the environmental impact, as light radiation is both clean energy and a clean reagent.

The most applied semiconductor in heterogeneous photocatalysis for water treatment is titanium dioxide, being efficient, chemically and biologically inert enough, and also cheap [2,3,4]. Unfortunately, due to its wide band gap, it can only utilize UV radiation, which is just a small fraction of sunlight (4–5%). Its three naturally occurring modifications are rutile, anatase, and brookite. At low temperatures, anatase is favored, whereas, at higher temperatures, rutile is formed, and brookite is not produced in large quantities [5].

The artificial production of TiO_2_ for photochemical purposes can be realized by various procedures, such as precipitation [6], solvothermal [7,8,9], various sol–gel [10,11,12], microemulsion [13,14], and electrochemical methods [15,16]. Gas-phase processes are mostly used to produce thin catalyst films [17,18]. Solid-phase synthesis has the advantage of being an environmentally friendly and solvent- and additive-free cost-effective alternative for many industrial processes. The mechanochemical process is based on mixing starting and modifying materials in powder form, followed by grinding, which can be dry or wet [19,20,21].

The activity of a catalyst is determined by a combination of several parameters, such as the production method, phase composition, particle size, and specific surface area. The band gaps for rutile, anatase, and brookite are 3.01, 3.20, and 3.13 eV, respectively [22,23,24,25]. Of the commercially available catalysts, the so-called Degussa P25 TiO_2_ (25 ± 5% rutile, 75 ± 5% anatase) has the highest photocatalytic activity and is currently the accepted standard for environmental technologies. In order to extend the absorption of the semiconductor towards the visible range, the catalyst can be modified with various organic or inorganic compounds (e.g., dyes and complexes)] or elements [26,27,28]. Depending on whether the modification is carried out on the semiconductor surface or by incorporation into the crystal lattice, one can speak of deposition or doping. Precious metals, such as silver, can be attached to the TiO_2_ surface by sol–gel processes, mechanical mixing, chemical and photochemical deposition, or precipitation reduction [29]. They accumulate the photogenerated electrons, thus reducing the probability of charge recombination. The activity of the modified catalyst also depends on the chemical properties of the substrate to be degraded. Some research groups have obtained significant improvements by silvering the catalyst when used for the photocatalytic degradation of organic acids, e.g., oxalic and formic acids [30,31], aspartic acid [32], and some aromatic compounds such as aniline [33] and 2-chlorophenol [34].

Nitrogen has been found to be one of the most promising modifiers; it can be incorporated into the TiO_2_ crystal lattice in two ways [35,36]: in substitutional position, replacing the oxygen, or in interstitial position. Nitrogen-modified catalysts were successfully applied for the degradation of various dyes [37,38,39] and phenol [40,41] under visible and UV light.

The combination of heterogeneous photocatalysis and ozonation significantly increased the decomposition rate of the model compounds, often resulting in a synergistic effect [42,43,44] because the electron capture by ozone and oxygen reduces the probability of the recombination of the photogenerated (e^−^, h^+^) pair, producing ^•^OH radicals. The amount of this species and H_2_O_2_ is increased upon UV irradiation of ozone.

**Figure 1:**
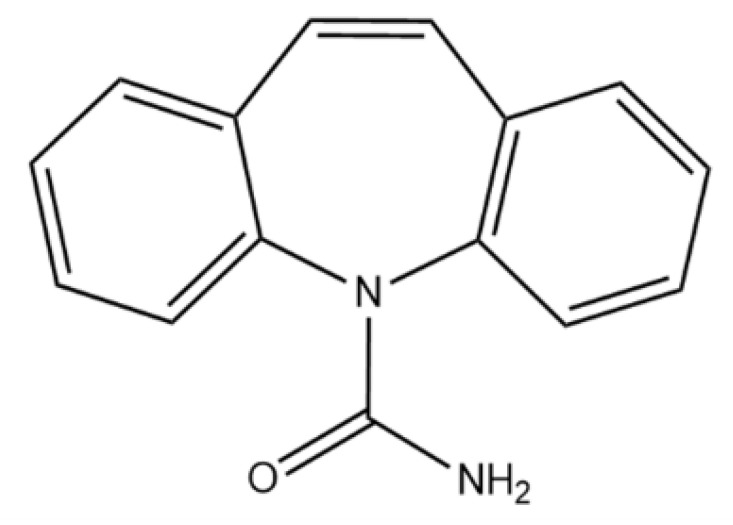
Structural formula of carbamazepine.

Carbamazepine (5H-dibenzo[b,f]azepine-5-carboxamide, CBZ, Figure 1) is marketed as an anticonvulsant and analgesic, widely used to treat trigeminal neuralgia, seizures, and other psychiatric disorders, and detected in significant concentrations in surface water, tap water, and wastewater [45]. In addition, it is difficult to degrade using conventional water treatment technologies [46,47,48].

Kowalska and co-workers successfully realized the solar-powered degradation of carbamazepine and other drug molecules with nitrogen-doped TiO_2_ immobilized on polystyrene spheres [48]. A g-C_3_N_4_/TiO_2_ composite photocatalyst was also used for this purpose by Wang et al., utilizing UV-Vis LED as a light source [49]. Sb_2_O_3_/TiO_2_ catalysts doped with neodymium (0–2%) were also applied for the degradation of carbamazepine, although under UVC irradiation [50].

Ozonation as another AOP was also used for CBZ decomposition, completed with the determination of several intermediates formed during this process [51,52]. The main intermediates were BQM (1-(2-benzaldehyde)-4-hydro-(1H,3H)-quinazoline-2-one), BaQM (1-(2-benzoic acid)-4-hydro-(1H,3H)-quinazolin-2-one), BaQD (1-(2-benzoic acid)-(1H,3H)-quinazoline-2,4-dione), and BQD (1-(2-benzaldehyde)-(1H,3H)-quinazoline-2,4-dione).

Mathew and Kanmani found that ozonation proved to be more efficient than heterogeneous photocatalysis for the removal of this compound [53], but they did not combine these two methods.

In this work, on the basis of previous results, our goal was the characterization of various TiO_2_ catalysts, both the standard Degussa P25 as a reference, and mechanochemically treated and modified (Ag- and/or N-doped) ones, with regard to their structure, morphology, and photocatalytic activity. Carbamazepine, which proved to be recalcitrant to conventional water treatment technologies, was chosen as a test compound for degradation under both UV and visible light. Photocatalytic treatment was also combined with ozonation, resulting in synergic effects. The identification of the intermediates formed under various conditions proved carbamazepine to be applicable for the elucidation of the degradation mechanisms, and, thus, for the characterization (and comparison) of photocatalysts regarding their activity and its relation to their structural and morphological properties. Our results indicated that, in the mineralization of carbamazepine, the importance of the different oxidizing radicals significantly deviate, and their generation strongly depends on both exciting light energy and the structure of the catalyst. Since the intermediates or end-products formed during the degradation of several biologically active compounds have a higher environmental impact than the starting compound does, toxicity measurements were also carried out during the course of irradiation. Our results indicated a similar situation with carbamazepine.

## 2. Results and Discussion

### 2.1. Physical Characterization of the Catalysts Prepared

Before the investigation of the photocatalytic behavior of our catalysts, their physical characterization was carried out in order to reveal the relations between them.

#### 2.1.1. Phase Composition and Specific Surface Area

**Figure 2:**
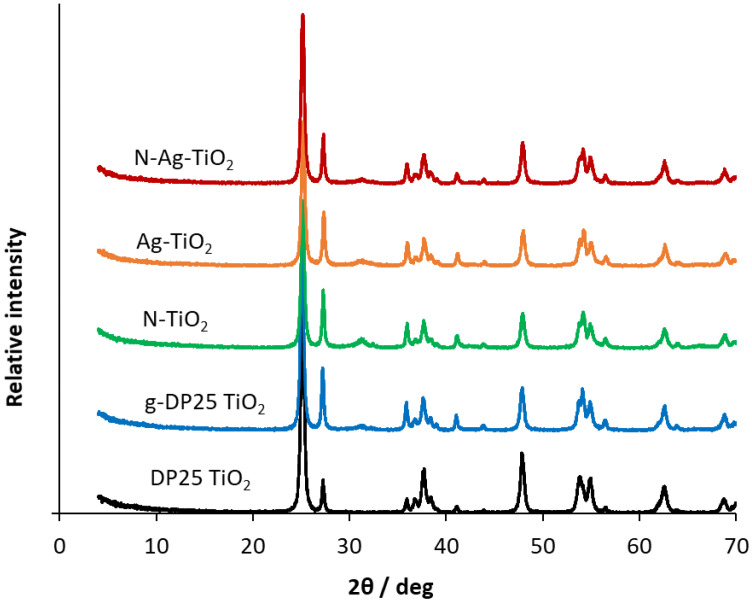
XRD patterns of pristine and modified DP25 TiO_2_ catalysts.

XRD measurements were carried out to determine the phase composition and the crystallite size of the pristine and mechanochemically modified DP25 TiO_2_ photocatalysts. In the sample names, “g” designates the ground (i.e., mechanochemically treated) DP25 to distinguish it from the untreated DP25. N and Ag in the names indicate doping with these elements (by mechanochemical treatment). The XRD patterns obtained for the catalysts samples studied are shown in Figure 2. The diffractograms were evaluated by using the 00–021-1272, 00–021-1276, 00-029-1360 Powder Diffraction File (PDF) of ICDD (International Centre for Diffraction Data) of anatase, rutile, and brookite, respectively. Figure S1 indicates the identification and quantification of the individual phases in samples DP25 and Ag-TiO_2_.

Table 1 and Figure S2 show the ratios of the three allotropic modifications of TiO_2_ (anatase, brookite, rutile—determined by XRD measurements) and the specific surface area values of the photocatalysts studied.

Whereas the commercially available DP25 TiO_2_ contains 88.6% anatase and 11.4% rutile, the ground DP25 TiO_2_ catalyst (g-DP25 TiO_2_) has its rutile content increased by 13.0%, its anatase content decreased by 26.2%, and brookite is present in 13.2%. Grinding significantly changed the phase composition of the catalyst. This is probably due to the local heating between the catalyst to be milled and the grinding balls, which promotes the phase transformation, i.e., the formation of thermodynamically more stable rutile. The modification with nitrogen inhibits the anatase conversion, whereas the silver-modified catalyst shows a significant decrease in the anatase content (58.5%) and an increase in both rutile and brookite. Notably, despite the changes in the phase composition upon mechanochemical treatment, the crystallite size (determined by XRD measurements) remained constant (28 ± 2 nm).

In all cases, the specific surface area of the catalysts is smaller than that of the starting catalyst (DP25 TiO_2_) (Table 1). Although there is not always a close correlation between specific surface area and catalyst efficiency, a larger specific surface area promotes surface adsorption of the reactants involved in the photo-induced processes, which is an important prerequisite for the reactions to proceed.

#### 2.1.2. Adsorption Measurements

**Figure 3:**
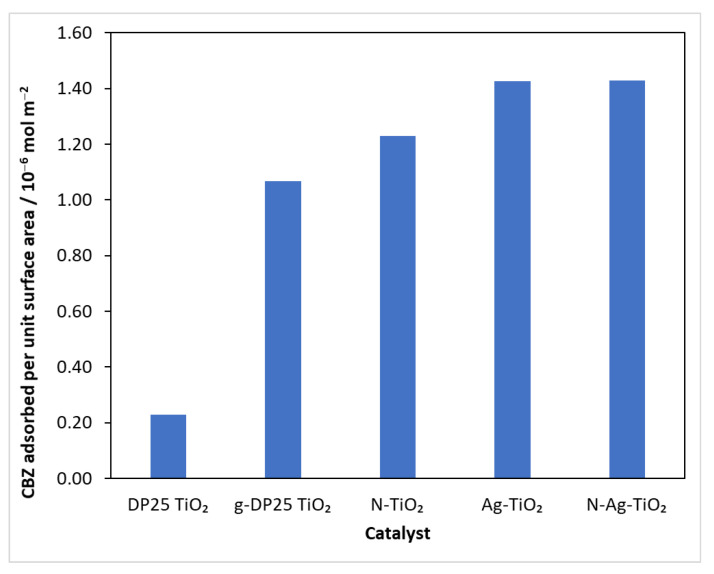
The amount of CBZ bound on a surface unit of catalysts.

At the beginning of the experiments, the reaction mixture was stirred in the reactor for 30 min without illumination to homogenize and achieve adsorption–desorption equilibrium, and was then sampled. Subtracting the concentration after the adsorption period from the initial value, the amount of model compound bound was calculated. Knowing the specific surface area of the catalysts (Table 1), the amount of carbamazepine adsorbed per unit surface area could be calculated (Figure 3).

The results show that, although the specific surface area of the catalysts prepared by grinding is smaller (Table 1), the amount of substrate bound per unit surface area of these catalysts was always higher than that for the reference (DP25 TiO_2_). This suggests that, during the mechanochemical process, the surface develops nodal points that favor the adsorption of the test compound.

#### 2.1.3. SEM and TEM Analysis

In order to gain some pieces of information regarding the particle size and elemental composition of the catalyst samples, SEM and TEM measurements were also carried out. Compared to the particle size of pristine DP25 TiO_2_ (Figure S3a,b), which is about the crystallite size, all of the mechanochemically treated samples display particles larger by more than two orders (Figure S3c–f), independently of the doping. The distribution of the particle size in the latter samples is very wide, where the largest particles exceed 10 μm, but much smaller pieces also occur in various fractions. In addition, the shapes of these particles are irregular, and frequently have sharp edges, deviating from those of the pristine DP25, which are spherical with a rather narrow size distribution. These results clearly indicate that mechanochemical treatment promoted the strong agglomeration of smaller particles, which also manifested in the decrease in the specific surface area (Table 1). The elemental compositions indicate the existence of the corresponding doping elements (Figure S3c–f) well, with lower fractions than those of Ti and O.

The results of the TEM measurements confirmed the unambiguous presence of silver in the Ag-DP25 catalyst (Figure S4). Based on the elemental maps, silver was only clearly detectable in Ag nanoparticles around a few tens of nm. Deviating from the SEM measurements, however, nitrogen could not be clearly detected because of the strong overlap of its signals with those of titanium.

#### 2.1.4. Band Gaps

The evaluation of the diffuse reflectance spectra for the prepared catalysts provided the band-gap energies (E_g_). As the data in Table S1 indicate, the band gaps of the samples prepared by mechanochemical treatment hardly differ from each other; their value is 3.02 ± 0.01 eV, independent of the doping elements. This is about 0.14 eV lower than that for the pristine DP25 TiO_2_ catalyst (3.16 eV). This drop in the band gap can be accounted for by the significant increase in the particle size resulting from the mechanical treatment. One may find a correlation between the rutile content and the band gap of the catalysts, but the alteration of the latter one is negligible compared to that of the phase composition. Instead, the rutile ratio is in much closer correlation to the nitrogen content as was also observed earlier [54].

### 2.2. Photoinduced Degradation of Carbamazepine

#### 2.2.1. Photolysis of Carbamazepine

For comparison, even if carbamazepine hardly absorbs in the wavelength ranges of the light sources used for photocatalytic experiments, if at all, its photolyses were also carried out under the same irradiation conditions. The concentration changes are shown in Figure S5.

Under UV light ((λ_max_) = 390 nm), the concentration of carbamazepine decreased by 2.67 mg dm^−3^ during 25 h of exposure, and its initial decomposition rate was 0.1 mg dm^−3^ h^−1^. The change in the concentration of the model compound during the other two illuminations was less than 0.15 mg dm^−3^. The compound only degrades significantly under UV light.

The change in the pH of the solution phase was also monitored. From a nearly identical starting value of 5.42–5.45, the largest increase was observed at visible light ((λ_max_) = 450 nm, ΔpH = 1.28). This result suggests that the intermediate formed during the minor degradation was basic in character and, thus, caused an increase in pH. The intermediates formed under UV light ((λ_max_) = 390 nm) were likely to be both acidic and basic in nature, and, therefore, the overall pH change was smaller. In accordance with this tendency, ΔpH was an intermediate value (0.88) at λ_max_ = 415 nm.

#### 2.2.2. Photocatalytic Degradation of Carbamazepine on Degussa P25 TiO_2_ Catalyst

**Figure 4:**
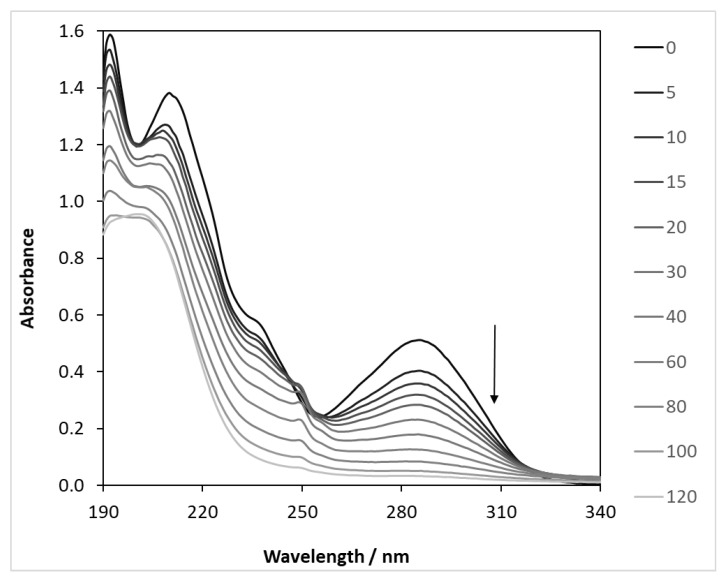
Light absorption changes during the photocatalysis of carbamazepine (λ(max) = 390 nm). c_(CBZ)0_: 10 mg dm^−3^, airstream: 10 dm^3^ h^−1^, 1 g dm^−3^ DP25 TiO_2_, l: 1 cm, λ_(det.)_: 285 nm.

The light absorption spectra of CBZ show two characteristic bands with maxima at 213 and 285 nm. By plotting the spectral variations in the illuminated samples, it can be clearly seen that the absorbance decreases at both wavelengths (Figure 4). The absorbance values measured at 285 nm were converted to concentration using a calibration curve, plotted as a function of the illumination time (Figure S6), and compared with the values determined by HPLC analysis.

The results clearly show that the concentrations are almost the same initially, but the difference between the two values increases over time. This is because absorbance is the sum of the absorbance of all of the components that absorb light at a given wavelength. However, it is simple and quick to measure and gives a good indication of the progress of the reaction. The exact concentration of the model compound was determined in all further cases by liquid chromatography analysis.

**Figure 5:**
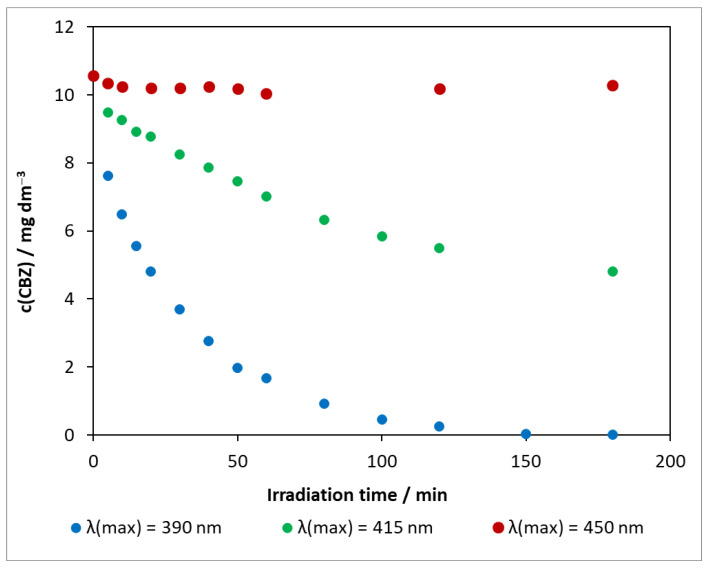
Changes in carbamazepine concentration for three light sources in the presence of DP25 TiO_2_ photocatalyst. c_(CBZ)0_: 10 mg dm^−3^, airstream: 10 dm^3^ h^−1^, 1 g dm^−3^ DP25 TiO_2_, λ_(det.)_: 285 nm.

The results obtained for the photocatalytic degradation of carbamazepine on DP25 TiO_2_ at various light sources are compared in Figure 5. Using the visible-light source of λ(max) = 450 nm, the concentration change in the presence of the Degussa P25 catalyst was minimal, and, during 3 h of exposure, was only 0.54 mg dm^−3^. Increasing the excitation light energy also increased the decomposition rate. When irradiated with an LED at λ(max) = 415 nm, the conversion was close to 50% in 3 h. Under UV light, the concentration of the model compound decreased to near the limit of detection in only 2 h. As observed in photolysis, the pH change was highest for the light source of λ(max) = 450 nm with ΔpH = 0.80, whereas, for the other two light sources, ΔpH_λ(max)= 390 nm_ = 0.60 and ΔpH_λ(max) = 415 nm_ = 0.65.

The presented series of experiments illustrate that the photoactivity of the reference catalyst varies significantly as a function of the excitation wavelength. The disadvantage of titanium dioxide is that it has a wide band gap and can therefore only utilize a small fraction of sunlight (4–5% UV).

#### 2.2.3. Degradation of Carbamazepine on Mechanochemically Modified Catalysts

**Figure 6:**
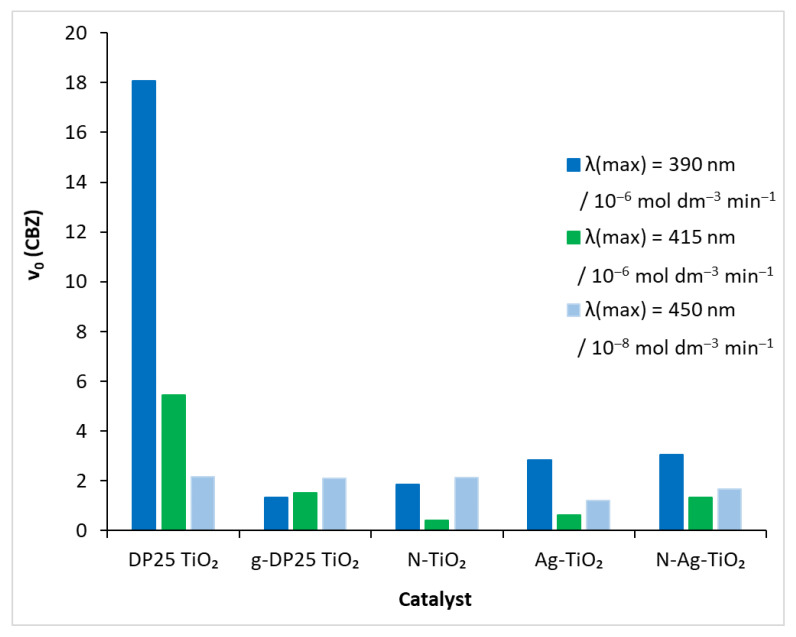
Initial decomposition rate of carbamazepine under different experimental conditions.

In order to obtain a catalyst that is active in visible light, the reference catalyst was modified by a mechanochemical process that also used nitrogen and silver. In each of the degradation experiments performed under different conditions (catalyst, light source), the time course of carbamazepine concentration was determined by HPLC analysis. The initial decomposition rate (v_0_) of the model compound was obtained by fitting a polynomial to this function (Figure 6). The values of the ‘rate-modifying’ effect (RME), i.e., initial decomposition rate on the actual catalyst/initial decomposition rate on the reference catalyst, calculated for the different catalysts, are shown in Table 2. Degussa P25 TiO_2_ in the presence of UV irradiation caused the compound to decompose rapidly (Figure 5): v_0_ = 18.07 × 10^−6^ mol dm^−3^ min^−1^. The efficiency of the process diminished as the light source energy decreased: for λ(max) = 415 nm, it was 5.46 × 10^−6^ mol dm^−3^ min^−1^, whereas, for λ(max) = 450 nm, it was two orders of magnitude lower (2.1 × 10^−8^ mol dm^−3^ min^−1^).

The photoactivity of the DP25 catalyst exposed to a 1 h mechanochemical treatment (designated as g-DP25 TiO_2_) decreased significantly in the UV range: RME = 0.07. This value increased gradually when reducing the irradiation light energy. In the visible range (at 450 nm), it approached the efficiency of the reference catalyst: RME = 0.97.

Grinding significantly changed the phase composition of the catalyst (Table 1). The decrease in the proportion of the anatase phase from 88.6% to 62.4% may be one of the reasons for the drastic change in photoactivity in the UV range. However, the higher fraction of the rutile phase (24.4% instead of 11.4%) and the appearance of brookite may explain the visible range efficiency almost reaching that of the reference.

Notably, the modification with nitrogen and silver increased the decomposition rate of the model compound compared to the case of the g-DP25 catalyst in the UV range (Figure 6). This is probably due to the decreased probability for the recombination photo-produced electron–hole pair in the modified catalysts, resulting in an increased lifetime of the active particles [29]. This is a general effect in the case of metal deposition or doping (mostly with precious metals such as Ag) due to the trapping of the photogenerated electrons by the metal particles or clusters. Hence, the positively charged holes more efficiently oxidize water molecules or hydroxide ions, producing hydroxyl radicals of a high oxidation potential [55]. Regarding nitrogen doping, however, increasing the valence-band potential enhances the probability of electrons reaching the conduction band upon excitation. Thus, an increased number of conduction-band electrons results in a more efficient production of superoxide (or hydroperoxyl) radicals via electron scavenging by dissolved oxygen molecules. In the case of the doubly modified titanium dioxide, N-Ag-TiO_2_, both effects operate, causing the highest degradation rate among the treated catalyst upon UV irradiation.

In the visible range (λ(max) = 415 nm), the beneficial effect of the nitrogen modification is shown by the fact that the photocatalytic efficiency approached that of DP25 and g-DP25, whereas silverization significantly lowered RME, even combined with N-doping. These results clearly indicate that the measure of the adsorption of CBZ on these catalysts is not in a close correlation with the efficiency of its photocatalytic degradation.

#### 2.2.4. Degradation of Carbamazepine by Photocatalysis Combined with Ozonation

**Figure 7:**
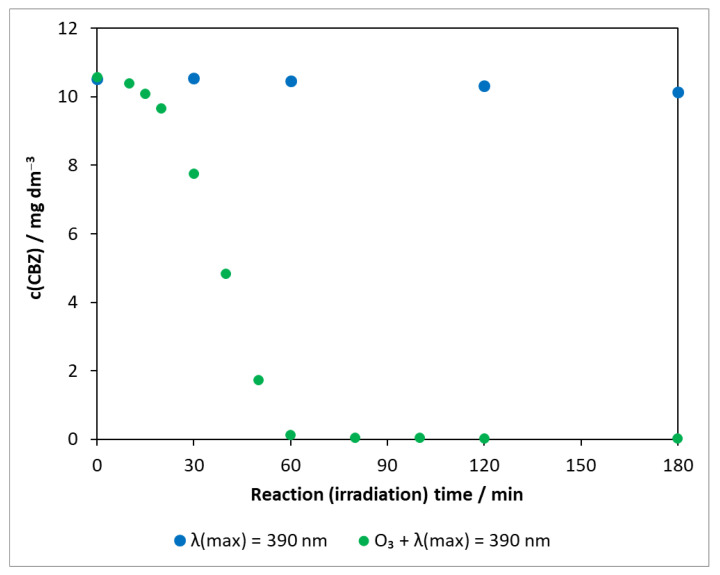
Changes in the concentration of carbamazepine under illumination and ozonation. c_(CBZ)0_: 10 mg dm^−3^, airstream: 10 dm^3^ h^−1^, λ_(det.)_: 285 nm, c(O_3_) = 0.35 mM min^−1^.

The combined use of two high-efficiency methods (AOPs), heterogeneous photocatalysis and ozonation, was also studied for the degradation of carbamazepine. In order to explore the role of ozone and to determine whether a synergistic effect occurs as reported in several pieces of literatures [51,52,53], experiments in the absence of photocatalysts were also carried out. The transformation of carbamazepine was investigated in the presence of ozone under UV irradiation (Figure 7).

As shown in Section 2.2.1, the concentration of carbamazepine in photolysis did not change significantly during 3 h of illumination (with a decay rate of 1.94 × 10^−9^ mol dm^−3^ min^−1^), even at 390 nm (Figure S5). For comparison, the corresponding points are also depicted in Figure 7. In the presence of ozone under UV irradiation, only a slight change was observed at the beginning; then, the concentration of the test compound decreased rapidly and got below the limit of detection after 60 min. The degradation rate was determined from 20 min onwards, ignoring the initial phase (1.67 × 10^−6^ mol dm^−3^ min^−1^).

**Figure 8:**
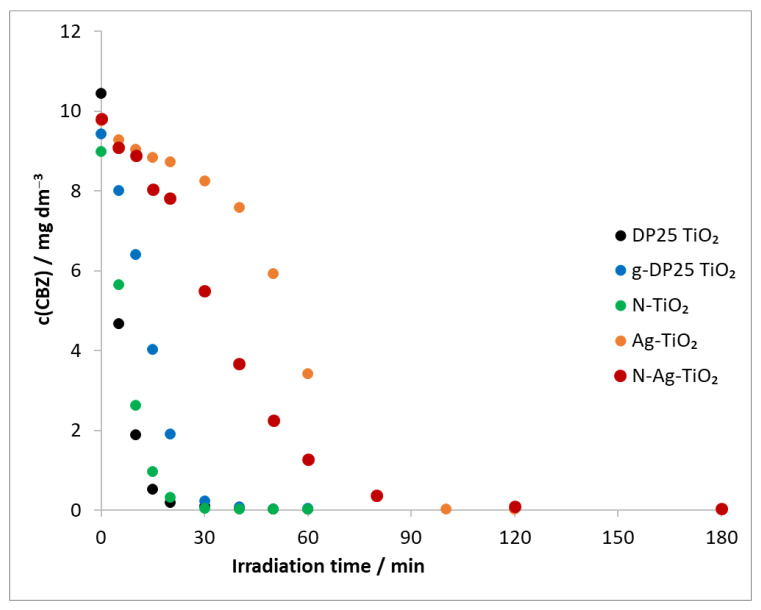
Temporal change in model compound concentration in heterogeneous photocatalysis (λ(max) = 390 nm) and ozonation. c_(CBZ)0_: 10 mg dm^−3^, airstream: 10 dm^3^ h^−1^, λ_(det.)_: 285 nm, c(O_3_) = 0.35 mM min^−1^.

The degradation of carbamazepine was also carried out by using a combination of all of the catalysts described previously, ozone, and a λ(max) = 390 nm light source (Figure 8).

Based on the determined initial degradation rates (in the first 20 min), the most efficient degradation was achieved in the presence of the DP25 TiO_2_ catalyst, followed by N-TiO_2_, g-DP25 TiO_2_, N-Ag-TiO_2_, and Ag-TiO_2_ in order of decreasing efficiency (Table 3, v_0_(TiO_2_ + UV + O_3_)). The decomposition rate of the model compound was higher for all catalysts when combining the two methods than when using heterogeneous photocatalysis alone (Table 3, v_0_(TiO_2_ + UV + O_3_)/v_0_(TiO_2_ + UV)). A 19-fold relative increase was observed for the N-TiO_2_ catalyst, whereas this value was much smaller for the other catalysts, decreasing in the order of g-DP25 TiO_2_, DP25 TiO_2_, N-Ag-TiO_2_, and Ag-TiO_2_.

The sum of the decomposition rates for the DP25 TiO_2_ + UV process (18.07 × 10^−6^ mol dm^−3^ min^−1^) and for the O_3_+UV method (1.67 × 10^−6^ mol dm^−3^ min^−1^) is 19.74 × 10^−6^ mol dm^−3^ min^−1^. This value can be considered as v_0, expected_ without any synergism (Table 4). In contrast, the reaction rate calculated from the concentration decrease in the combined method was 51.78 × 10^−6^ mol dm^−3^ min^−1^. This implies that a synergistic effect occurs, i.e., each process amplifies the effect of the other.

Similar calculations were made for the other catalysts (Table 4). An overview of these results clearly shows that no synergistic effect was observed in reaction mixtures containing the Ag-TiO_2_ catalyst. In the presence of N-Ag-TiO_2_, the efficiency of the combined process was only slightly higher (5.05 × 10^−6^ mol dm^−3^ min^−1^) than the sum of the individual processes (4.71 × 10^−6^ mol dm^−3^ min^−1^). However, the decomposition rate of the model compound significantly increased when N-TiO_2_ and ozonation were used together.

#### 2.2.5. Change in COD in Photocatalysis

**Figure 9:**
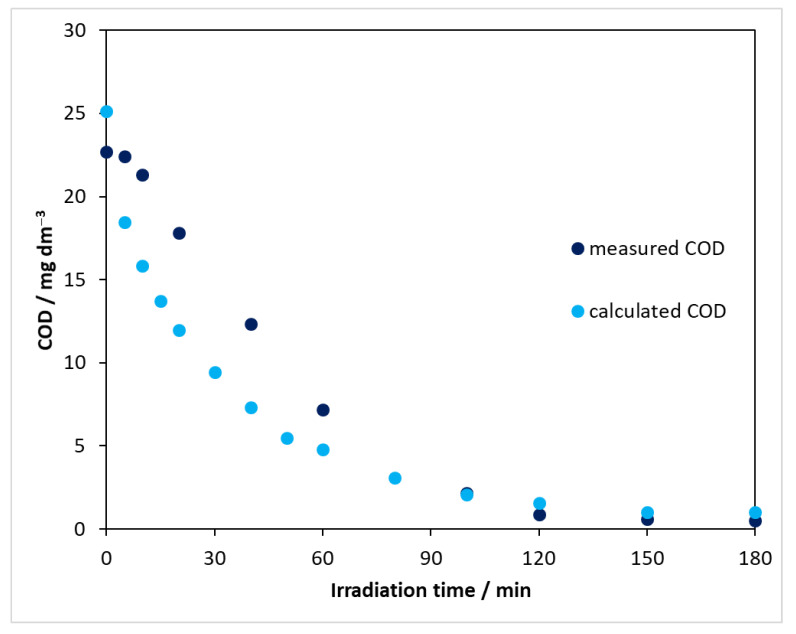
Comparison of COD_meas_ and COD_CBZ_ using DP25 TiO_2_ catalyst and λ(max) = 390 nm light source.

In monitoring the processes taking place in redox processes, an important parameter is the chemical oxygen demand (COD), which provides information on the oxidation state of the compounds to be degraded, i.e., whether the system contains any further oxidizable components. The COD values of solutions of carbamazepine at different concentrations were determined to obtain a calibration curve. Using this, the exact concentration of CBZ could be used to determine the COD that carbamazepine would give if it were present in the solution alone (COD_CBZ_). Figure 9 clearly shows that the COD value measured during the degradation was lower than the corresponding COD_CBZ_. This means that the oxidation state of the intermediates produced from carbamazepine was higher than that of the parent compound. It can also be seen that the intermediates formed were also gradually transformed and degraded as the reaction proceeded, with virtually no further oxidizable compound in the reaction mixture at the end of the process.

The chemical oxygen demand was also determined for each sample in heterogeneous photocatalytic experiments in the presence of modified catalysts; a decrease in COD was observed for each catalyst during the illumination (Table S2). In addition, after 180 min, the decrease in COD for the modified catalysts was smaller than that for the reference catalyst in all cases. The results are in good agreement with the previous ones, i.e., the photocatalytic decomposition efficiency decreased when using the modified catalysts.

The combination of heterogeneous photocatalysis with ozonation significantly increased the degradation rate of carbamazepine as shown in Table 3, also demonstrating a synergistic effect in most cases (Table 4). This observation suggested that such a combination could be applicable for the total mineralization of CBZ within a reasonable time under these experimental conditions. This possibility has been proved by COD measurements. The COD values collected during the combined treatments applying UV irradiation (Figure S7) indicate that ozonation coupled with heterogeneous photocatalysis, in the case of DP25 and N-TiO_2_ catalysts, resulted in a practically total mineralization of CBZ within 60 or 80 min, respectively, due to the strong synergistic effects.

#### 2.2.6. Ecotoxicity

**Figure 10:**
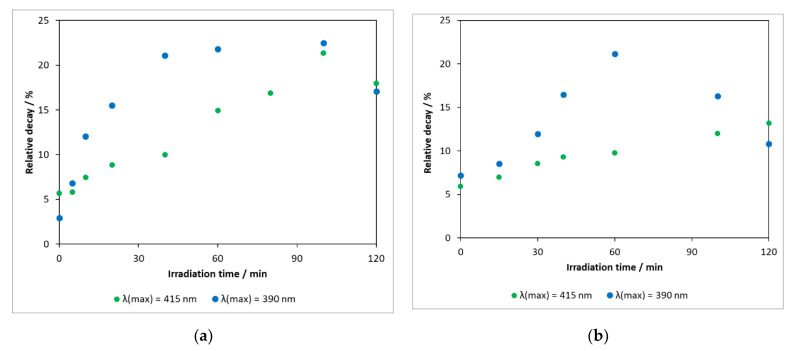
(**a**) Changes in toxicity measured during heterogeneous photocatalytic degradation of carbamazepine on catalyst Degussa P25 TiO_2_ and (**b**) N-TiO_2_.

Pharmaceutical compounds released into the environment have the potential to alter the feeding and reproductive habits of living organisms and the functioning of their basic biological systems necessary to sustain life. It is therefore important to know the ecotoxicity of the parent compound and the intermediates formed during its degradation. In our work, toxicity measurements were carried out by the application of luminescent *Vibrio fischeri* bacteria. Figure 10a shows that, in the heterogeneous photocatalytic treatment using the photocatalyst DP25 TiO_2_ (λ(max) = 390 nm), the toxicity of the reaction mixture increased gradually with time, whereas the concentration of the starting compound decreased significantly (reaching the limit of detection in 2 h, Figure 5). This means that the toxicity of the intermediates formed is more significant for *Vibrio fischeri* bacteria than that of the parent compound. The decrease in toxicity after 90 min was probably due to the degradation of the intermediates.

Reducing the energy of the irradiation light (λ(max) = 415 nm) significantly modified the activity of the catalyst (about 55% of the model compound was converted in 120 min Figure 5), and the formation of toxic intermediates was also a slower process.

A similar finding was made by Donner and co-workers when investigating the ecotoxicity of carbamazepine intermediates formed by UV photolysis [56]. These results indicate that a complete mineralization of the intermediates ought to be achieved to avoid an increased toxicity.

Toxicity measurements were also carried out for the illuminated samples in experiments performed in the presence of a nitrogen-modified catalyst with a lower photoactivity (Figure 10b). An interesting and noteworthy result was that the maximum relative degradation of the bacteria (light source: λ(max) = 390 nm) was 21.2% (60 min), which was almost identical to the 22.5% (40–100 min) observed for the DP25 TiO_2_ catalyst. This was presumably due to the formation of intermediates with significant toxicity during degradation in both cases.

Similar trends were observed in measurements with other modified catalysts. For low-energy light sources, the toxicity increased gradually with the exposure time. This is consistent with the fact that carbamazepine did not completely decompose within 3 h, due to the lower photoactivity, and the rate of the formation of (toxic) intermediates was higher than their decomposition rate. As the energy of the light source increased, the activity of the catalysts increased, too, resulting in the decomposition of the intermediates, which led to a decrease in the toxic effect.

If UV illumination was also used in the presence of ozone, the toxicity of the samples started to already increase at the beginning of the illumination (Figure S8). The maximum value reached 40%. A similarly high maximum was observed for the combination of heterogeneous photocatalysis and ozonation, but at a much shorter irradiation time, after 10–20 min of exposure, and then decreased steeply, probably due to the rapid formation and degradation of intermediates. Notably, if ozone was applied alone, the maximum toxicity approached 80%.

#### 2.2.7. Formation and Detection of Intermediates

When carbamazepine was degraded under different conditions, an HPLC analysis of the samples could be used to not only determine the actual concentration of the model compound but also to obtain information on the changes in the amount of intermediates formed.

In the case where carbamazepine was degraded by ozonation alone, for comparison, four peaks appeared in the HPLC chromatograms at retention times (tR) of 4.20; 4.70; 5.10, and 5.65 min. Of these, the area under the peak of the compound with tR = 5.10 min was 394,820 mAU min, whereas the other three intermediate compounds were much smaller (about 1000 mAU min, Figure S9). Notably, the peak areas did not correspond directly to the absolute concentrations because the sensitivity of the detector in the HPLC equipment was not the same for all compounds detected. Hence, the peak areas represent relative concentrations, which cannot be quantitatively compared. Nevertheless, the compound-specific sensitivities (e.g., based on molar absorbances in the case of the photometric detector) did not significantly deviate; therefore, the peak areas could be utilized for semi-quantitative comparisons, especially in the case of time-dependent relative concentration profiles. The compounds were identified by HPLC-MS in positive ion mode. The compound with a retention time of 5.10 min had an *m/z* value of 251. In view of the results in the literature, it corresponds to 1-(2-benzaldehyde)-4-hydro-(1H,3H)-quinazoline-2-one (BQM), which is a metabolite of the model compound [51] and also an intermediate in its photocatalytic decomposition [52] (Figure S10a).

In all of the experiments using ozone, an emitting intermediate was monitored (excitation: 285 nm, emission: 420 nm). Plotting the measured emission intensities together with the sub-peak areas determined from the HPLC analysis, it was clear that the emitting intermediate was BQM (Figure S11).

The variation over time in the amount of BQM determined by chromatography shows a good agreement with the relative decay of the bacteria (Figure S8). This result suggests that BQM is likely to have a significant toxicity to *Vibrio fischeri* bacteria (Figure S12). Of course, the toxicity of other (minor) intermediates cannot be excluded. Notably, the compounds present in the treated samples proved to be stable even after 30 days.

**Figure 11:**
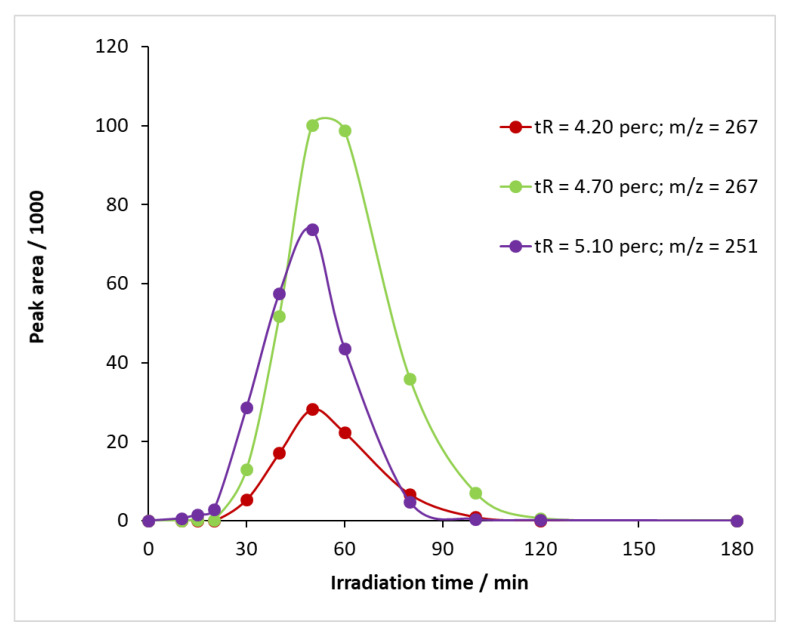
Temporal change in the relative concentration of intermediates formed during the reaction of carbamazepine with ozone and UV light.

When UV illumination was applied in the presence of ozone, not only BQM but also other intermediates were formed from carbamazepine (Figure S13) at retention times of 4.20 and 4.70 min, and the time variation in their sub-peak areas is shown in Figure 11. The *m/z* value of the compounds detectable at 4.20 and 4.70 min was 267 (Figure S10b,c). By studying the literature, two intermediates can be assigned to this *m/z* value: 1-(2-benzaldehyde)-(1H,3H)-quinazoline-2,4-dione (BQD) and 1-(2-benzoic acid)-4-hydro-(1H,3H)-quinazolin-2-one (BaQM) (Figure S10d). For the determination of the exact structure of the compounds associated with each retention time, HPLC-MS/MS studies are required, which have not been carried out at this stage of the research.

Using ozone alone, the BQM had a maximum peak area of 394,820 mAU min, and the measured relative degradation of bacteria was 77.5% (Figures S8 and S11). When illuminated with UV light, the BQM peak area became much smaller at 33,046 mAU min, and the relative degradation of the bacteria was 39.5%. The peak area of the intermediate (and, hence, its amount) was reduced to about one-tenth, and the toxicity of the reaction mixture was reduced to about half. These data indicate that the other compounds that formed, such as BQD and BaQM, also inhibit the biological activity of *Vibrio fischeri* bacteria.

When combining heterogeneous photocatalysis and ozonation, the highest efficiency increase was observed in the presence of N-TiO_2_ (Table 3 and Table 4). Besides BQM, in this system, other intermediates appeared in the chromatogram of the sample illuminated for 15 min on this catalyst, but with a much lower intensity (Figure S14). The intermediate with a retention time of 5.60 min has an *m/z* value of 258 and can be assigned to 2,2’-imino-dibenzoic acid (Figure S15a) based on the literature. The other new intermediate that appears has a retention time of 7.00 min, an *m/z* value 196, and is suggested in the literature to be acridone (Figure S15b) [51,52].

These results provide useful information for exploring the processes involved in the heterogeneous photocatalysis discussed earlier.

In Table S3, the intermediates formed in suspensions containing the Degussa P25 catalyst are compared, designated by their retention times. The results regarding irradiations at different wavelength indicate that an increasing amount of intermediates appeared as the illumination energy was increased:

λ(max) = 450 nm—2, λ(max) = 415 nm—3, whereas λ(max) = 390 nm—6.

Compounds eluting at 5.65 and 5.45 min are characterized by 253 *m/z*, corresponding to carbamazepine hydroxylated at different sites (Figure S16).

Under UV irradiation, the intermediates formed rapidly, reaching their maximum amount (corresponding peak area) after only 5–30 min of irradiation, and then decomposed. After 180 min, the concentrations of virtually all components fell below the detection limit, i.e., complete mineralization was achieved (Figure S17).

**Figure 12:**
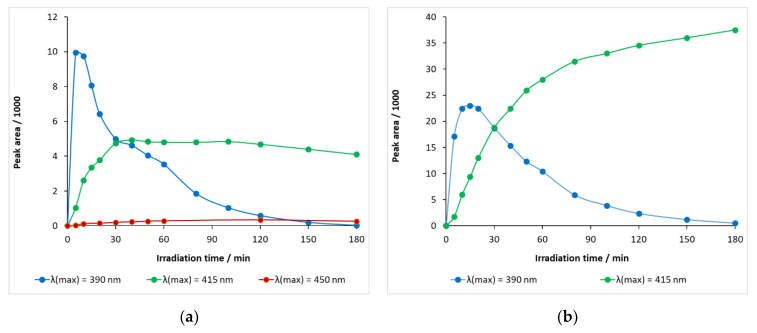
Temporal change in the relative concentrations of hydroxy-carbamazepine (*m/z* 253) at retention times (**a**) 5.65 min and (**b**) 5.45 min in carbamazepine solution containing DP-25 TiO_2_.

Hydroxylated carbamazepine with a retention time of 5.65 min—λ(max) = 390 nm—was rapidly generated, peaking at 5–10 min and then decreasing at a considerable rate (Figure 12a). When the light source energy was decreased—λ(max) = 415 nm—, the rate of formation became significantly slower and the decay, after 40 min, was very slow, indicating that both formation and decomposition took place competitively in this period of time. When the wavelength of the light source was further increased (to λ(max) = 450 nm), a very modest increase in the relative concentration of the hydroxylated intermediate was observed, with the maximum peak area being less than 400 mAU min (Figure 12a).

The maximum peak area of a hydroxylated carbamazepine with a retention time of 5.45 min at λ(max) = 390 nm was 22,987 mAU min (15 min) and then gradually decayed (Figure 12b). When λ(max) = 415 nm LED was used, it monotonously accumulated during the illumination time, probably with a formation rate much higher than the decay rate, with a peak area of 37,512 mAU min at 180 min.

The BQM intermediate, with a retention time of 5.10 min, thus typical of ozone experiments, also appeared in DP25 TiO_2_ catalyst suspensions during 390 nm illumination, but its peak area was much smaller, even smaller than that of the hydroxylated carbamazepine, and decreased after 15 min. It appeared to be a rapidly transforming intermediate.

On all of the studied photocatalysts, intermediates formed with the following retention times: 4.70; 5.10; 5.45; 5.65, and 6.00 min. Their relative concentration profiles as functions of time are displayed in Figures S18 and S19.

The intermediate with a retention time of 4.70 min—BaQM or BQD—was formed in small amounts in the presence of the DP25 TiO_2_ catalyst and decomposed after 15–20 min. In contrast, in other cases—g-DP25 TiO_2_, N-TiO_2_, N-Ag-TiO_2_—it was formed after a short induction period and its concentration increased gradually, especially in suspensions containing N-Ag-TiO_2_. Decomposition in the presence of N-TiO_2_ was observed after 90 min (Figure S18a). The “toxic” BQM intermediate, with a retention time of 5.10 min, was formed in the presence of N-TiO_2_ and g-DP25 TiO_2_ in higher amounts than in the other suspensions (Figure S18b). This explains the results obtained in our toxicity studies (Figure 10b), according to which, photocatalysis samples in N-TiO_2_-containing suspensions showed a relatively high toxicity, although the conversion of carbamazepine was less than in the presence of DP25 TiO_2_.

In all cases, the peak area of hydroxycarbamazine detectable at 5.45 min was relatively large (Figure S19). In the presence of DP25 TiO_2_, the amount decreased after 15 min, i.e., the decomposition process became dominant. In the other experiments, this occurred later if at all: N-TiO_2_—40 min, g-DP25 TiO_2_—60 min, N-Ag-TiO_2_—120 min, Ag-TiO_2_—only formation.

The peak area of hydroxycarbamazepine with a retention time of 5.65 min in suspensions containing modified catalysts was also relatively large, and it was rapidly transformed in the system catalyzed by DP25 TiO_2_ (Figure S19). In the presence of Ag-TiO_2_, it was formed in higher amounts than the 5.45 min isomer, whereas, in N-Ag-TiO_2_ suspensions, the difference in formation kinetics between the two isomers was smaller.

Regarding the application of irradiation with λ(max) = 415 nm for each catalyst, not all intermediates were present in all cases, so: DP25 TiO_2_—2, g-DP25 TiO_2_—4, N-TiO_2_—5, Ag-TiO_2_—2, and N-Ag-TiO_2_—4 different intermediates were identified (Table S4). Using a light source of λ(max) = 450 nm, fewer intermediates could be detected (Table S5), and their peak areas were small. This is not surprising, since the decrease in carbamazepine concentration was also small. In both cases, only the formation of intermediates was observed without their decay due to the low photocatalytic efficiencies.

**Figure 13:**
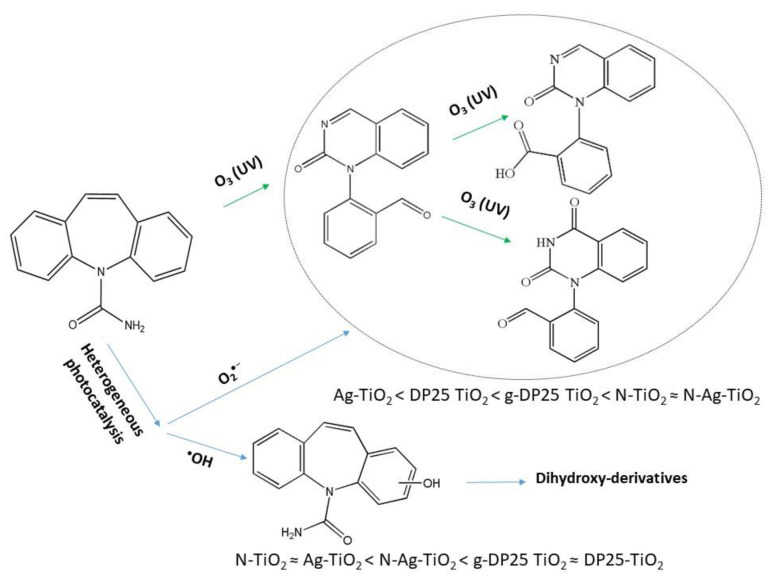
Suggested degradation routes of carbamazepine under various conditions. The efficiency orders regarding transformations via reactions with O_2_^•−^ or HO^•^ are also given for the photocatalysts studied.

Summarizing the results presented above, the formation–decomposition tendency of intermediates varies depending on the catalyst used. In general, among the identified intermediates, in suspensions containing DP25 TiO_2_, the formation of hydroxylated carbamazepine is favored, and the other compounds only appear for a short time with a low intensity during the degradation of carbamazepine. In contrast, in the presence of N-TiO_2_, conditions for the formation of BQM intermediates are similar to those in ozonized systems, whereas hydroxylation is of less importance. This result is in good agreement with the earlier observation that modification with nitrogen promotes the formation of the superoxide radical ion [57]. On the basis of the detected intermediates, the main degradation routes of the carbamazepine under various conditions are shown in Figure 13. In the presence of ozone and nitrogen-doped photocatalysts, the formation of the ring-opened intermediates was preferred, indicating the role of superoxide radical anion, whereas hydroxy-derivatives were predominantly produced by bare titanium dioxide due to the efficient generation of hydroxyl radicals.

## 3. Materials and Methods

### 3.1. Materials

The following commercially available analytical grade chemicals were used without further purification: CH_3_CN (gradient purity, ≥99.9%), formic acid (≥95%) from VWR International Kft. (Debrecen, Hungary), Degussa P25 TiO_2_, carbamazepine, urea, and AgNO_3_, from Sigma-Aldrich Kft. (Budapest, Hungary). Compressed air was introduced (via bubbling) into the reaction mixtures from a gas bottle [54]. Freeze-dried bacteria (for Lumistox bacteria test) were provided by Hach Lange GmbH (Düsseldorf, Germany).

### 3.2. Characterization

The specific surface area was determined by nitrogen adsorption/desorption isotherms measured with a Micromeritics ASAP 2000-type instrument on samples (weight ≈ 1.0 g) previously outgassed in vacuum at 160 °C. The surface areas of the samples were determined by the Brunauer–Emmett–Teller (BET) method from the corresponding nitrogen adsorption isotherms.

The XRD patterns were obtained on a Philips PW 3710 type powder diffractometer (Philips Analytical, Almelo, The Netherlands) with a Cu-Kα radiation source (λ = 1.5405 Å). The crystallite size and phase composition were determined by X-ray diffraction measurement (Philips PW3710, Cu Kα radiation, 50 kV, and 40 mA).

Data collections were carried out with X’Pert Data Collector software (v.: 2.0e). The full width at half-maximum (FWHM) values of the individual reflections were determined by the profile-fitting treatment of the X’Pert High Score Plus software (v.: 5.0). The peak broadening caused by the samples was explained by the presence of very small crystallites. The broadening of the 101 and 200 reflections of anatase (measured breadth minus the instrumental breadth) was used to calculate the average crystallite size by the well-established Scherrer equation. The FWHM values of the 110 and 211 reflections of rutile (of the Degussa P25 sample heated at 1000 °C), respectively, were employed as the instrumental breadth. The 00-021-1272, 00-021-1276, 00-029-1360 Powder Diffraction File (PDF) of ICDD (International Centre for Diffraction Data) of anatase, rutile, and brookite, respectively, were used to identify phases.

Diffuse reflectance spectra (DRSs) were recorded on a luminescence spectrometer (LS 50-B, PerkinElmer, Waltham, MA, USA) equipped with an integrating sphere attachment, and BaSO_4_ was used as a reference standard. The band-gap energy was calculated using Tauc plot of square of the Kubelka–Munk function against photon energy [54].

The microstructure/morphology of the samples was tested by FEI/ThermoFisher Apreo S scanning electron microscope. Observation by SEM was carried out in high vacuum mode with an accelerating voltage of 20.0 kV. In order to acquire the best resolution for imaging, the samples were fixed onto a carbon-based adhesive tape. Energy dispersive X-ray (EDX) spectra were collected at 20.0 kV (EDAX AMETEK Octane Elect Plus).

A Talos F200X G2 instrument (Thermo Fisher, Waltham, MA, USA) equipped with a field-emission gun and a four-detector Super-X energy-dispersive X-ray spectrometer was used at 200 kV for TEM and elemental analysis. Scanning transmission electron microscopy techniques (high-angle annular dark-field imaging and EDS elemental mapping) and bright-field and high-resolution TEM imaging were used to visualize nanoparticles.

### 3.3. Photochemical Experiments

The photolysis and photocatalysis experiments were carried out using a laboratory-scale 2 cm thick cylindrical reactor [55] with a volume of 80 cm^3^, made of Duran glass. Gas (air/ozone) was introduced through one of the septum-lined stubs at a flow rate of 10 dm^3^ h^−1^. The supplied air provided both continuous mixing and oxygen for the process. Homogenization of the reaction mixture was also facilitated by magnetic stirring.

For ozone decomposition, ozone was produced from air flowing at 10 dm^3^ h^−1^ using a LAB2B laboratory ozone generator (Ozone Engineering, El Sobrante, CA, USA). Its concentration was determined by absorption of the gas in potassium iodide solution, and the amount of iodine released was titrated with sodium thiosulphate.

The light source was placed behind the reactor at a distance of 10 cm. The temperature of the reaction mixture did not change significantly during the illuminations, rising by a total of 3–4 °C over 180 min. The light sources used were characterized by the following parameters (Figure S20):

Light source 1 (blue): λ(max) = 390 nm;

λ(range) = 370–430 nm, power: 60 W, flux density: 60 mW cm^−2^;

Light source 2 (green): λ(max) = 415 nm;

λ(range) = 390–450 nm, power: 50 W, flux density: 90 mW cm^−2^;

Light source 3 (red): λ(max) = 450 nm;

λ(range) = 410–750 nm, power: 50 W, flux density: 60 mW cm^−2^.

The concentration of catalyst used in all experiments was 1 g dm^−3^. The catalysts modified by grinding settled in the reactor despite the stirring and gas flow, and the particles agglomerated were treated by ultrasonic treatment. One day before illumination, 80 mg of catalyst was added to 10 cm^3^ of distilled water, and the suspension was placed in an ultrasonic bath for 30 min to reduce the size of the aggregates and increase homogeneity. Then, the suspension was stirred overnight because ultrasonication of water generates hydroxyl radicals, which may react with the model compound before illumination, causing an error.

The suspension prepared was added the next day to the solution containing the test compound. The reaction mixture was stirred in the reactor for 30 min without illumination to achieve proper homogenization and adsorption–desorption equilibrium. During the irradiation, 5 cm^3^ samples were taken at fixed intervals depending on the reaction rate. A Millipore Millex-LCR 0.45 μm filter was used to separate the solid phase from the liquid.

### 3.4. Analysis of Samples

#### 3.4.1. Acquisition of Light Absorption and Emission

Changes in light absorption of the reaction mixture were recorded by a Scinco S3100 UV/Vis spectrophotometer (Scinco C. Ltd., Seoul, Republic of Korea) using a quartz cuvette with an optical path length of 1 cm. The measurement range was 200–1000 nm in all cases. The emission and excitation spectra were obtained with a Perkin Elmer LS 50B (PerkinElmer, Waltham, MA, USA) spectrofluorometer.

#### 3.4.2. HPLC Analysis

To determine the actual concentration of the model compound and to separate and identify the intermediates formed, the illuminated samples were analyzed by reverse phase liquid chromatography. Measurements of carbamazepine and intermediates were performed using a Shimadzu LC-20AD liquid chromatograph (Shimadzu, Kyoto, Japan) (HPLC-1). The HPLC-MS (HPLC-2) separations and identification of intermediates were performed on an Acquity UPLC^TM^ system (Waters, Milford, MA, USA) equipped with a binary solvent delivery pump, an autosampler, a column heater block, a photodiode array detector (PDA), and a single quadrupole mass detector (QDa) with electrospray ionization. MS ionization was performed in positive ion mode. Eluents were generated using acetonitrile (ACN, VWR International, Radnor, PA, USA), formic acid (Carlo Erba reagents, Emmendingen, Germany), and ultrapure (type 1) water purified with a Simplicity^®^ water purification system (Millipore, Darmstadt, Germany). Eluent A was 5 *v/v*% ACN and 0.1 *v/v*% formic acid in water, and eluent B was 0.1 *v/v*% formic acid in ACN. For the separations, a Kinetex C18 (100 × 3 mm, 2.6 µm, 100 Å) analytical column (Phenomenex, Torrance, CA, USA) packed with core–shell silica stationary phase was utilized.

The flow rate of the eluents was 0.5 mL/min. During the elution, the initial 100% eluent A was held for 3 min; then, eluent A was decreased to 10% in 10 min and held there for 1 min. The column temperature was 40 °C and the injection volume was 2 µL. The detection wavelength was set to 285 nm.

Using the instrument available in our research group (HPLC-1), carbamazepine could be detected with a retention time of 6.50 ± 0.05 min. With the mass spectrometer coupled apparatus (HPLC-2), the retention time of the model compound was 4.71 ± 0.05 min. The same column was used for the measurements, so the difference is due to differences in instrumentation, mainly determined by the volume outside the column. For the identification of intermediates, the chromatograms obtained with the mass spectrometer coupled to the apparatus were related to our measurements: the retention times determined with the HPLC-2 liquid chromatograph (HPLC-2 measured) were divided by the ratio of the dead times of the two systems (HLPC-2 calculated), and the resulting chromatogram was compared with the chromatogram of HPLC-1 (Figure S21). After correction, it was possible to assign the mass spectra measured with HPLC-2 to the corresponding retention time intermediates.

#### 3.4.3. Toxicity Measurements

The toxicity was measured by using *Vibrio fischeri* luminescent bacteria. The sample preparation for antibacterial study is described in the Supplementary Material as Text S1 [58]. The luminescent intensity of *Vibrio fischeri* was detected by a Toxalert 100 device. The inhibition rate of bioluminescence could be achieved by Equation (1).

(1) $$Relative\;decomposition_{t}\left(\%\right)=\frac{I_{reference\left(t\right)}-I_{sample\left(t\right)}}{I_{reference\left(t\right)}}\times 100$$

where *I_reference(t)_* is the emission intensity of the reference or blind sample and *I_sample(t)_* is the emission intensity of the actual sample.

## 4. Conclusions

Besides the usual structural, morphological, and optical characterization, the photocatalytic activity of various titanium-dioxide-based semiconductors was also determined by the degradation of carbamazepine, an antidepressant. Importantly, this organic com-pound proved to be versatile for featuring the photoinduced reactions of the catalysts; it could be utilized to elucidate the production of different oxidizing radicals (^•^OH and HO_2_^•−^), strongly depending on the excitation energy and the composition of the catalysts. In addition, the intermediates identified in the decomposition of this test compound provided useful pieces of information regarding the reaction mechanism, also promoting the application of carbamazepine for the characterization of the oxidative degradation ability of such photocatalysts. Increasing the mineralization efficiency of photocatalysis by combination with ozonation can also be very important if the intermediates formed during the decomposition are more toxic than the original pollutant as proved in this case.

## Figures and Tables

**Table 1:** Phase composition and specific surface area (BET) of catalysts used.

	Anatase [%]	Rutile [%]	Brookite [%]	BET [m^2^ g^−1^]
DP25 TiO_2_	88.6	11.4	0.0	50.0
g-DP25 TiO_2_	62.4	24.4	13.2	37.3
N-TiO_2_	73.4	16.6	10.0	45.3
Ag-TiO_2_	58.5	25.8	15.7	38.6
N-Ag-TiO_2_	66.0	21.2	12.9	42.5

**Table 2:** Summary of the degradation rate modification effects (RME) relative to the reference catalyst at various irradiation wavelengths.

v_0, catalyst_/v_0, DP25 TiO2_	g-DP25 TiO_2_	N-TiO_2_	Ag-TiO_2_	N-Ag-TiO_2_
λ(max) = 390 nm	0.07	0.10	0.16	0.17
λ(max) = 415 nm	0.28	0.07	0.11	0.24
λ(max) = 450 nm	0.97	0.98	0.56	0.77

**Table 3:** Initial reaction rates for decompositions using the combined method.

v_0_ [10^−6^ mol dm^−3^ min^−1^]	DP25 TiO_2_	g-DP25 TiO_2_	N-TiO_2_	Ag-TiO_2_	N-Ag-TiO_2_
v_0_(TiO_2_ + UV + O_3_)	51.78	10.81	34.95	3.88	5.05
v_0_(TiO_2_ + UV + O_3_)/v_0_(TiO_2_ + UV)	2.87	8.07	18.99	1.37	1.66

**Table 4:** Demonstration of the synergistic effects.

v_0_ [10^−6^ mol dm^−3^ min^−1^]	DP25 TiO_2_	g-DP25 TiO_2_	N-TiO_2_	Ag-TiO_2_	N-Ag-TiO_2_
v_0, expected_	19.74	3.01	3.51	4.51	4.71
v_0_(TiO_2_ + UV + O_3_)	51.78	10.81	34.95	3.88	5.05

## Data Availability

The data presented in this study are available on request from the corresponding author. The data are not publicly available due to privacy.

## Supplementary Materials

The following supporting information can be downloaded at: https://www.mdpi.com/article/10.3390/molecules27228041/s1, Figure S1: (a) Identification and quantification of anatase and rutile in the XRD pattern of pristine DP25 TiO_2_, (b) Identification and quantification of anatase, rutile, and brookite in the XRD pattern of mechanochemically silverized DP25 TiO_2_. Figure S2: The phase composition of catalysts used. Figure S3: (a) SEM morphology of pristine DP25 TiO_2_ catalyst at higher magnification, (b) SEM morphology of pristine DP25 TiO_2_ catalyst at lower magnification, (c) SEM morphology and elemental composition of ground DP25 TiO_2_ catalyst, (d) SEM morphology and elemental composition of Ag-TiO_2_ catalyst, (e) SEM morphology and elemental composition of N-TiO_2_ catalyst, (f) SEM morphology and elemental composition of Ag-N-TiO_2_ catalyst. Figure S4: TEM analysis of the Ag-TiO_2_ catalyst; (a,b) TEM micrographs with different magnifications; (c–f) element maps obtained in STEM–EDS mode. Table S1: Band gaps of catalysts produced by grinding. Figure S5: Changes in carbamazepine concentration during photolysis. c_(CBZ)0_: 10 mg dm^−3^, airstream: 10 dm^3^ h^−1^. Figure S6: Changes in carbamazepine concentration using DP25 TiO_2_ photocatalyst, c_(CBZ)0_: 10 mg dm^−3^, airstream: 10 dm^3^ h^−1^, 1 g dm^−3^ DP25 TiO_2_, l: 1 cm, λ_(det.)_: 285 nm. Table S2: Comparison of changes in COD measured during illuminations. Figure S7: Changes in COD in carbamazepine solution during various treatments. Figure S8: Changes in toxicity in carbamazepine solution using ozone in various combinations. Figure S9: Chromatogram for the degradation of carbamazepine with ozone at 60 min reaction time. Figure S10: (a) Mass spectrum of the compound with tR = 5.1 min in the sample of CBZ ozonized for 60 min, and the structural formula of BQM (251 *m/z*), (b) Mass spectrum of the compound (*m/z* 267) with tR = 4.2 min in the sample of CBZ ozonized and irradiated for 50 min. (c) Mass spectrum of the compound (*m/z* 267) with tR = 4.7 min in the sample of CBZ ozonized and irradiated for 50 min. (d) Structural formula of (a) BQD (*m/z* 267) and (b) BaQM (*m/z* 267). Figure S11: Peak areas and emission intensities of BQM during ozonization of carbamazepine. Figure S12: Peak areas of BQM and change in toxicity during ozonization of carbamazepine. Figure S13: Chromatogram for the degradation of carbamazepine with ozone and UV light at 50 min reaction time. Figure S14: Fifteen min chromatogram for the degradation of carbamapezin, using N-TiO_2_ + O_3_ + UV. Figure S15: Structural formula of (a) 2,2’-imino-dibenzoic acid (*m/z* 258) and (b) acridone (*m/z* 196). Table S3: Intermediates formed under irradiation at various wavelength in the presence of DP25 catalyst. Figure S16: General structural formula of hydroxy-carbamazepine (*m/z* 253). Figure S17: Temporal changes in the relative concentrations of intermediates of retention times (a) 5.45, 5.56, and 6.00 min and (b) 4.71, 5.10, and 7.20 min upon UV irradiation of carbamazepine solution on DP25 TiO_2_ catalyst. Figure S18: Temporal changes in the relative concentrations of intermediates of retention time (a) 4.70 min and (b) 5.10 min upon UV irradiation of carbamazepine solution on various catalysts. Figure S19: Temporal changes in the relative concentrations of intermediates of retention time (a) 5.45 min and (b) 5.75 min upon UV irradiation of carbamazepine solution on various catalysts. Table S4: Intermediates formed under irradiation at λ(max) = 450 nm in the presence of various catalysts. Figure S20: Emission spectra of the light sources applied. Figure S21: Relating the results of two HPLC systems to each other. Text S1: Sample preparation for antibacterial study.
