# Supplementary material for: Characterization of Various Titanium-Dioxide-Based Catalysts Regarding Photocatalytic Mineralization of Carbamazepine also Combined with Ozonation
Source: Molecules. 2022 Nov 19;27(22):8041. doi: 10.3390/molecules27228041 (PMC9697621; doi:10.3390/molecules27228041)
Supplement: Supplementary file 1 [file molecules-27-08041-s001.zip › molecules-2018034-supplementary.pdf]

Supplementary Materials

for

Characterization of Various Titanium-Dioxide-Based Catalysts Regarding Photocatalytic Mineralization of Carbamazepine also Combined with Ozonation

Gábor Kocsis [1], Erzsébet Szabó-Bárdos [1], Orsolya Fónagy [1], Evelin Farsang [2], Tatjána Juzsakova [3], Miklós Jakab [4], Péter Pekker [5], Margit Kovács [1] and Ottó Horváth [1,*]

[1] Environmental and Inorganic Photochemistry Research Group, Center for Natural Sciences, University of Pannonia, P.O. Box 1158, H-8210 Veszprém, Hungary
[2] Analytical Chemistry Research Group, Center for Natural Sciences, University of Pannonia, P.O. Box 1158, H-8210 Veszprém, Hungary
[3] Sustainability Solutions Research Lab, Research Center for Biochemical, Environmental and Chemical Engineering, University of Pannonia, P.O. Box 1158, H-8210 Veszprém, Hungary
[4] Department of Materials Engineering, Research Center for Engineering Sciences, University of Pannonia, H-8210 Veszprém, P.O. Box 1158, Hungary
[5] Environmental Mineralogy Research Group, Research Institute of Biomolecular and Chemical Engineering, University of Pannonia, H-8210 Veszprem, P.O. Box 1158, Hungary

* Correspondence: horvath.otto@mk.uni-pannon.hu; Tel.: +36-88-624-000 (ext. 6049)

Content **Page Nr.**

Content	Page Nr.
Figures S1a,b	3
Figure S2	4
Figures S3a,b	5
Figure S3c	6
Figure S3d	7
Figure S3e	8
Figure S3f	9
Figure S4, Table S1	10
Figures S5, S6	11
Table S2, Figure S7	12
Figures S8, S9	13

Figures S10a,b	14
Figures S10c,d	15
Figures S11, S12	16
Figures S13, S14	17
Figure S15, Table S3, Figure S16	18
Figures S17, S18	19
Figure S19,Table S4	20
Figures S20, S21	21
Text S1	22

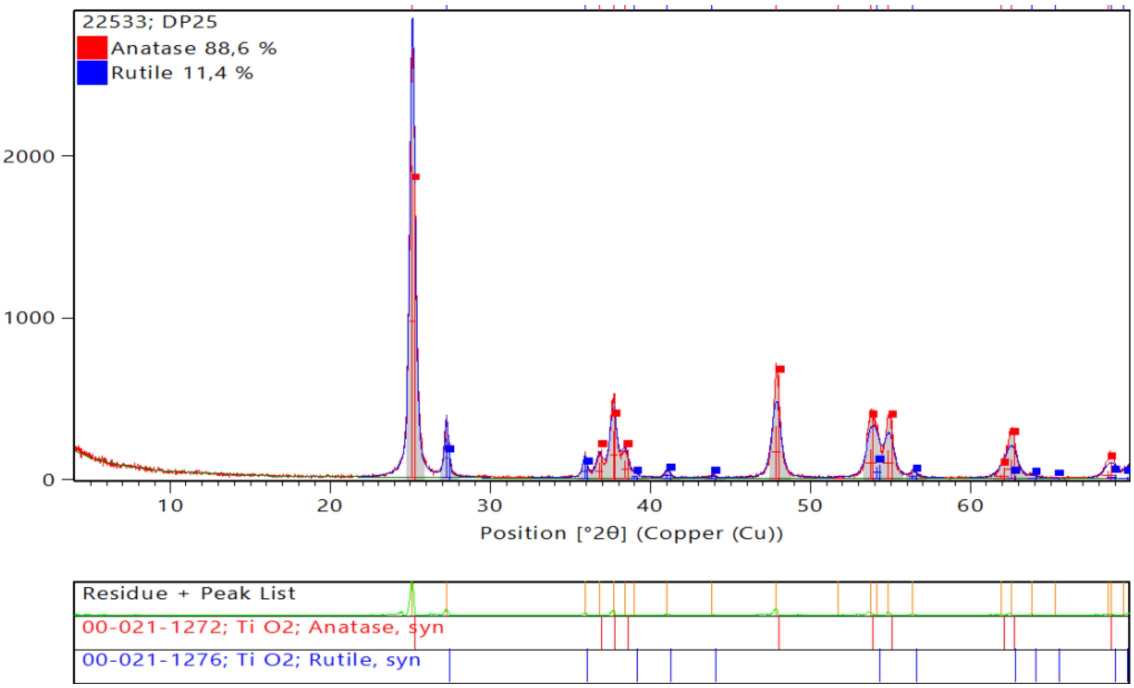

Figure S1. a) Identification and quantification of anatase and rutile in the XRD pattern of pristine DP25 TiO_2.

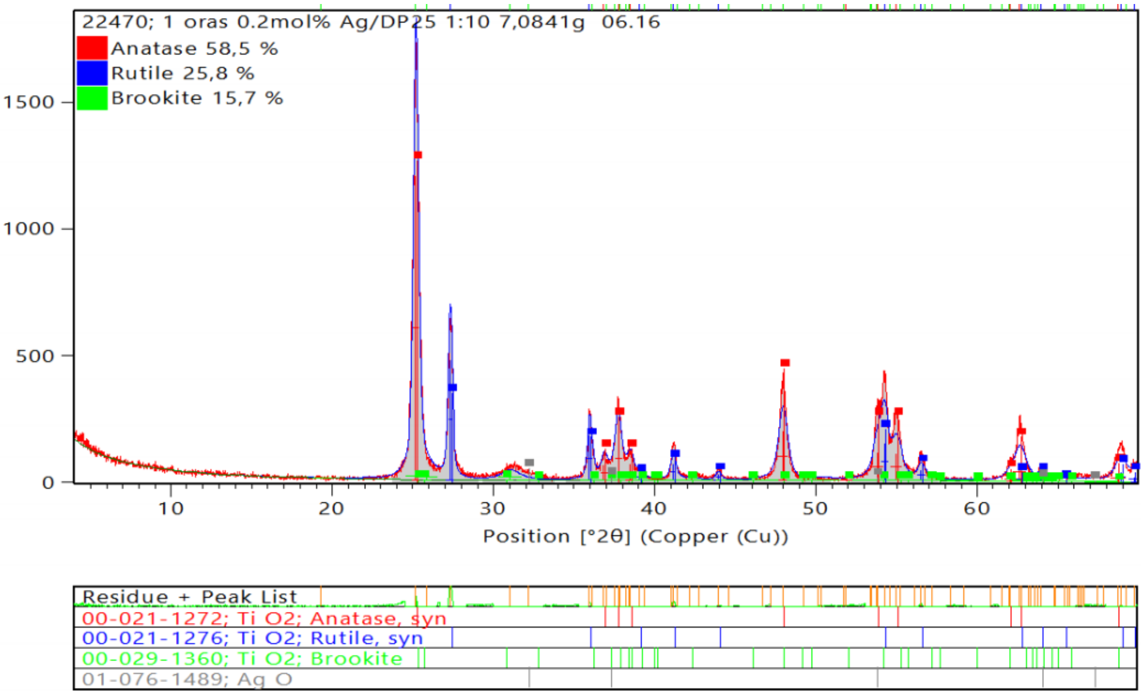

Figure S1. b) Identification and quantification of anatase, rutile, and brookite in the XRD pattern of mechanochemically silverized DP25 TiO_2.

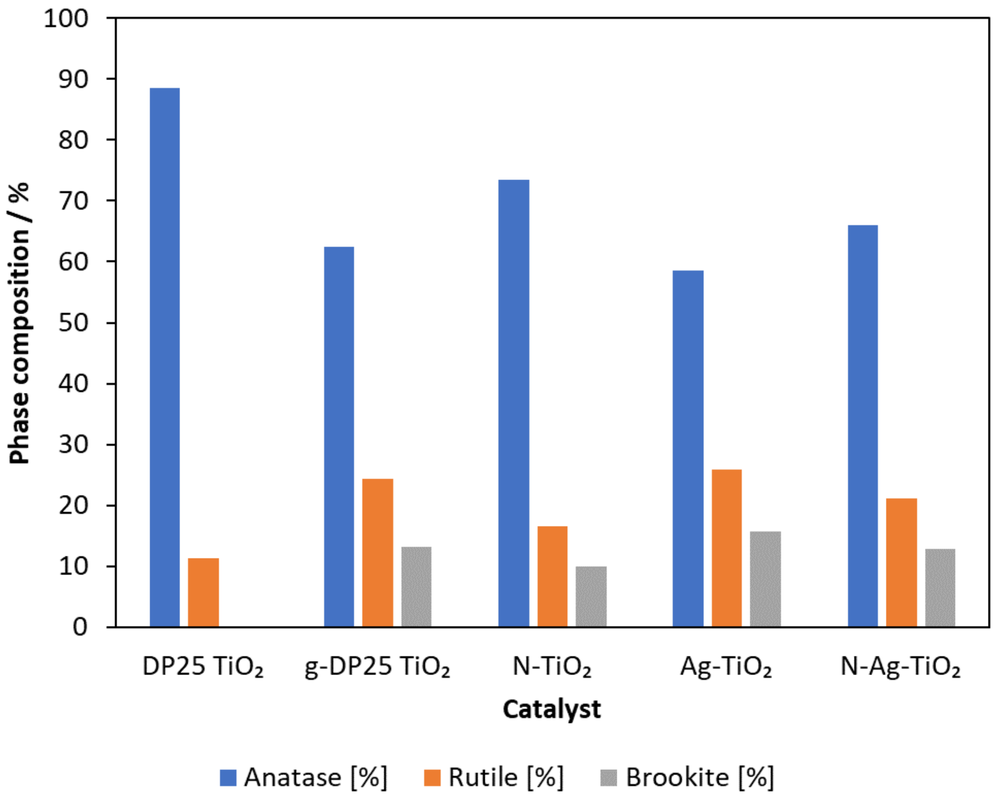

Figure S2. The phase composition of catalysts used.

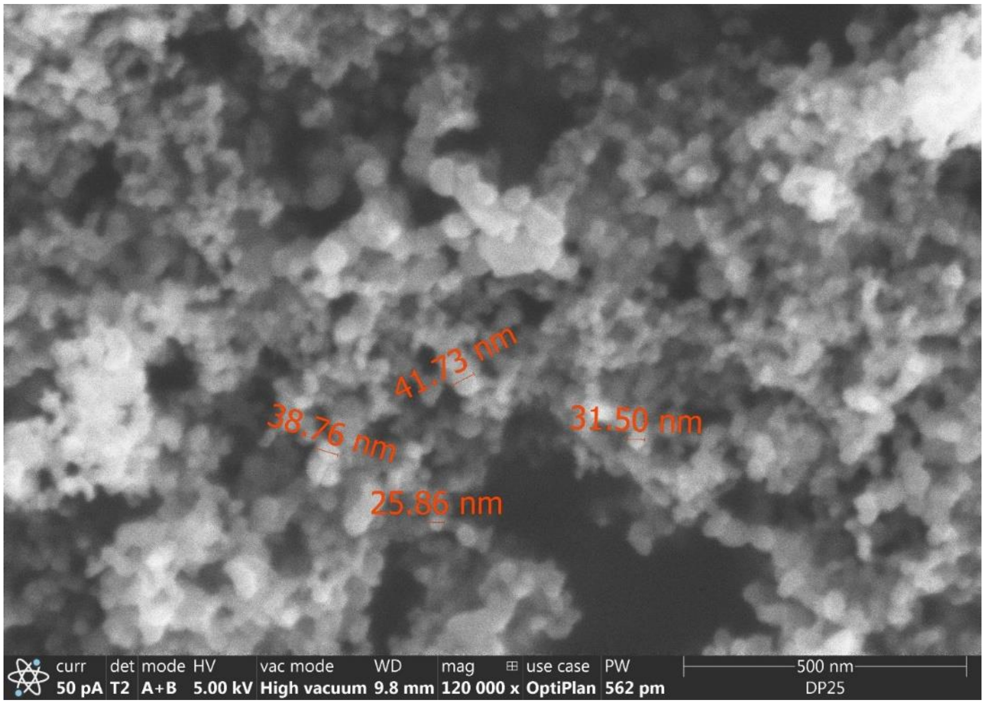

Figure S3. a) SEM morphology of pristine DP25 TiO_2 catalyst at higher magnification.

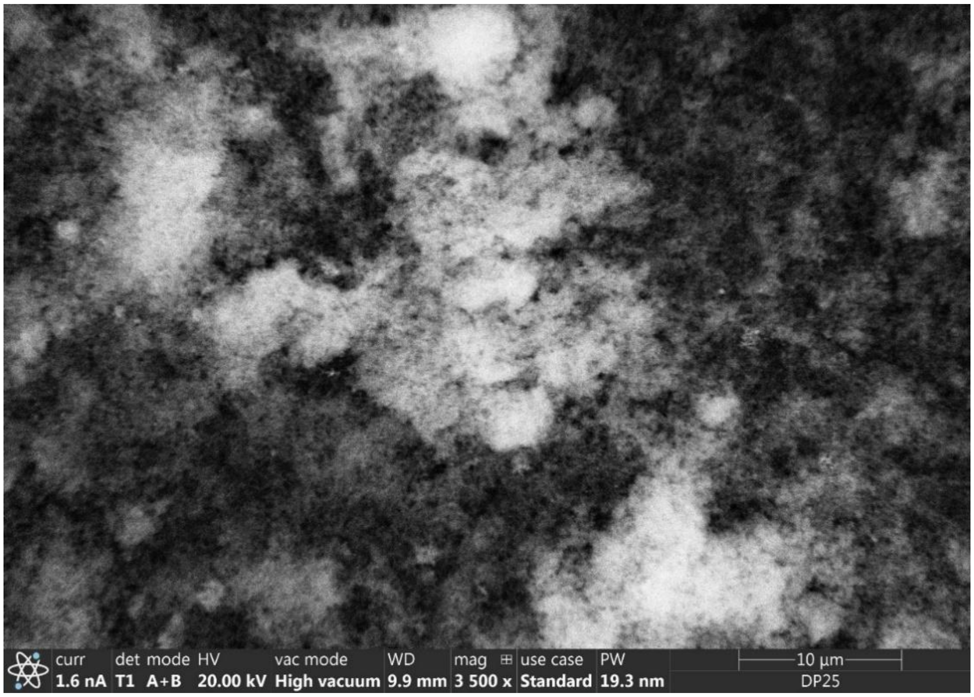

Figure S3. b) SEM morphology of pristine DP25 TiO_2 catalyst at lower magnification.

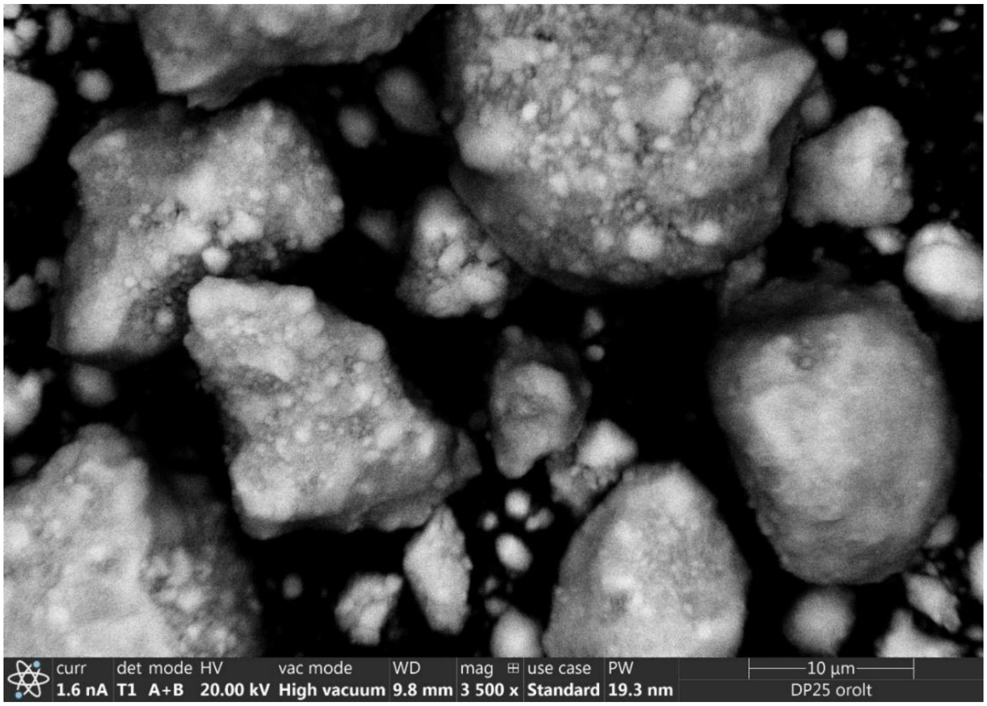

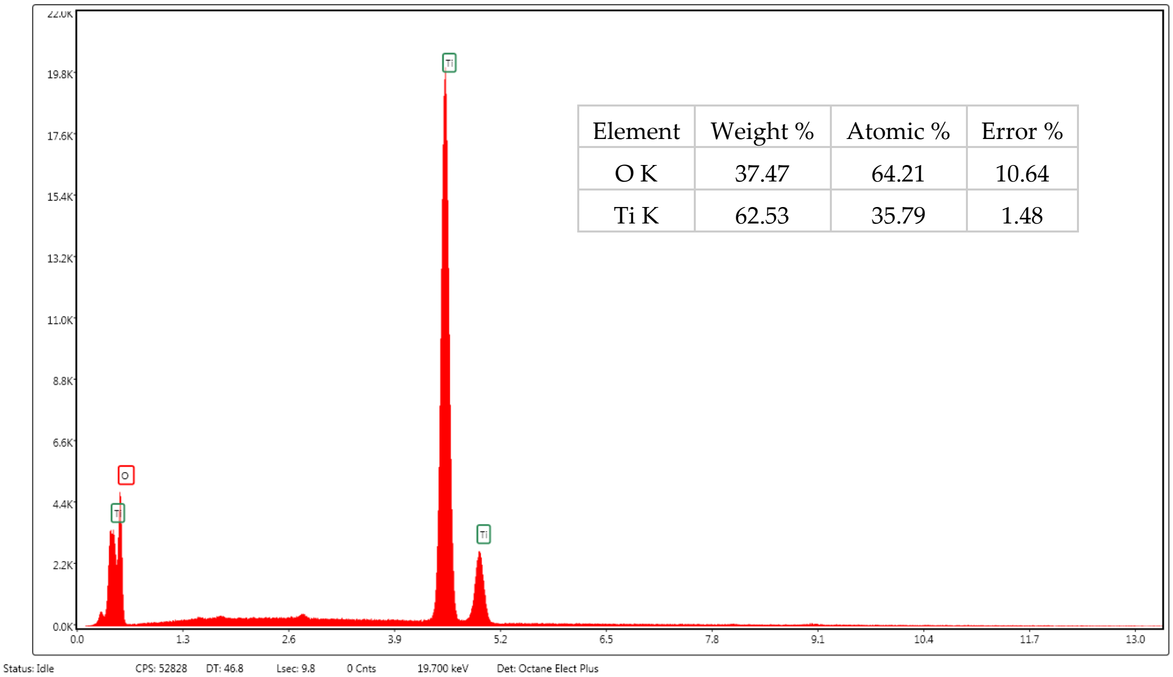

Element	Weight %	Atomic %	Error %
O K	37.47	64.21	10.64
Ti K	62.53	35.79	1.48

Figure S3. c) SEM morphology and elemental composition of ground DP25 TiO_2 catalyst.

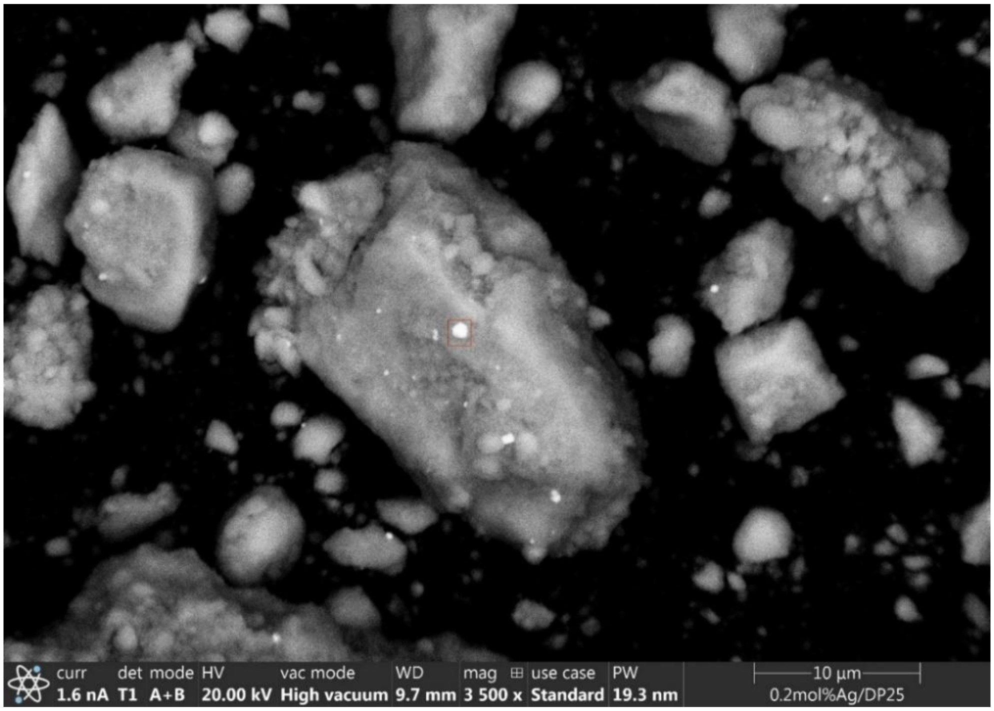

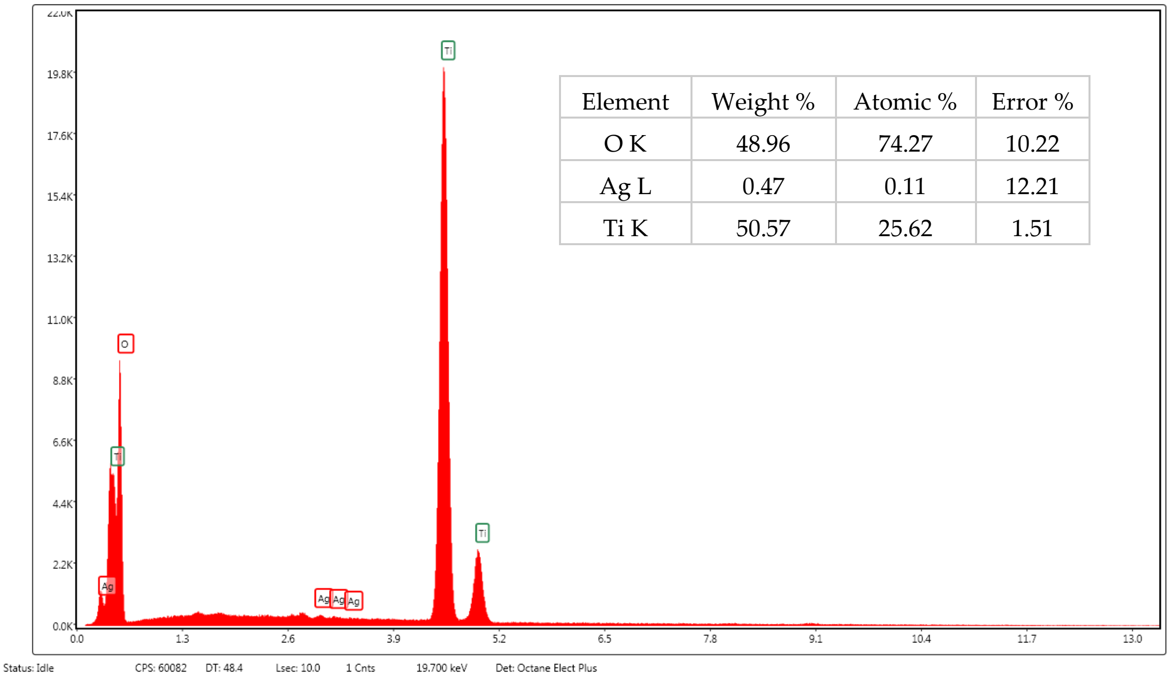

Element	Weight %	Atomic %	Error %
O K	48.96	74.27	10.22
Ag L	0.47	0.11	12.21
Ti K	50.57	25.62	1.51

Figure S3. d) SEM morphology and elemental composition of Ag-TiO_2 catalyst.

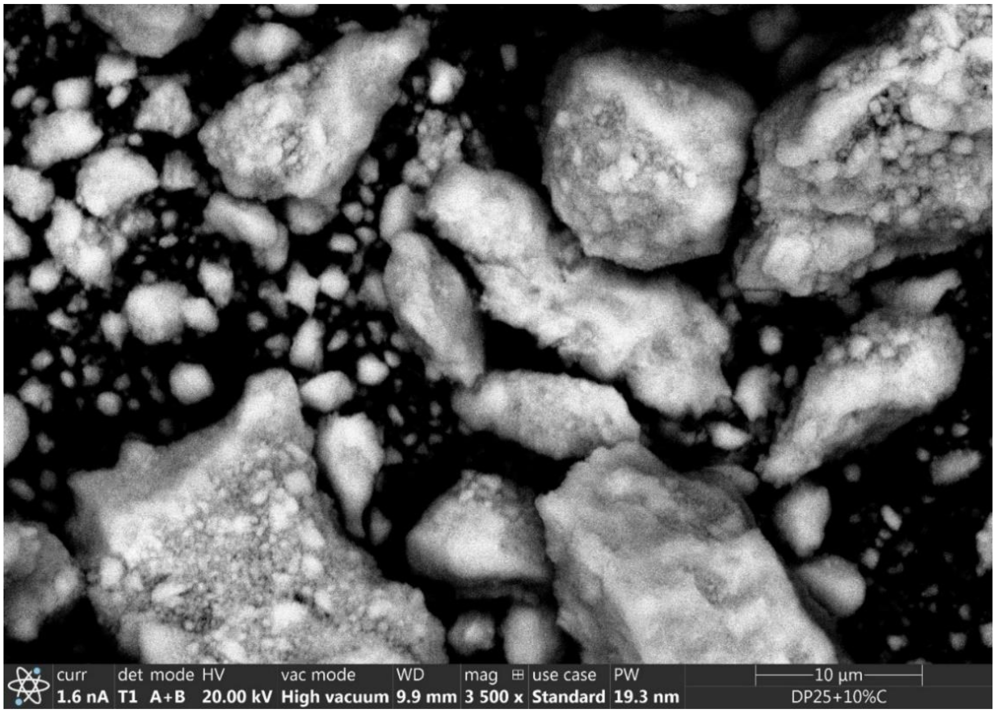

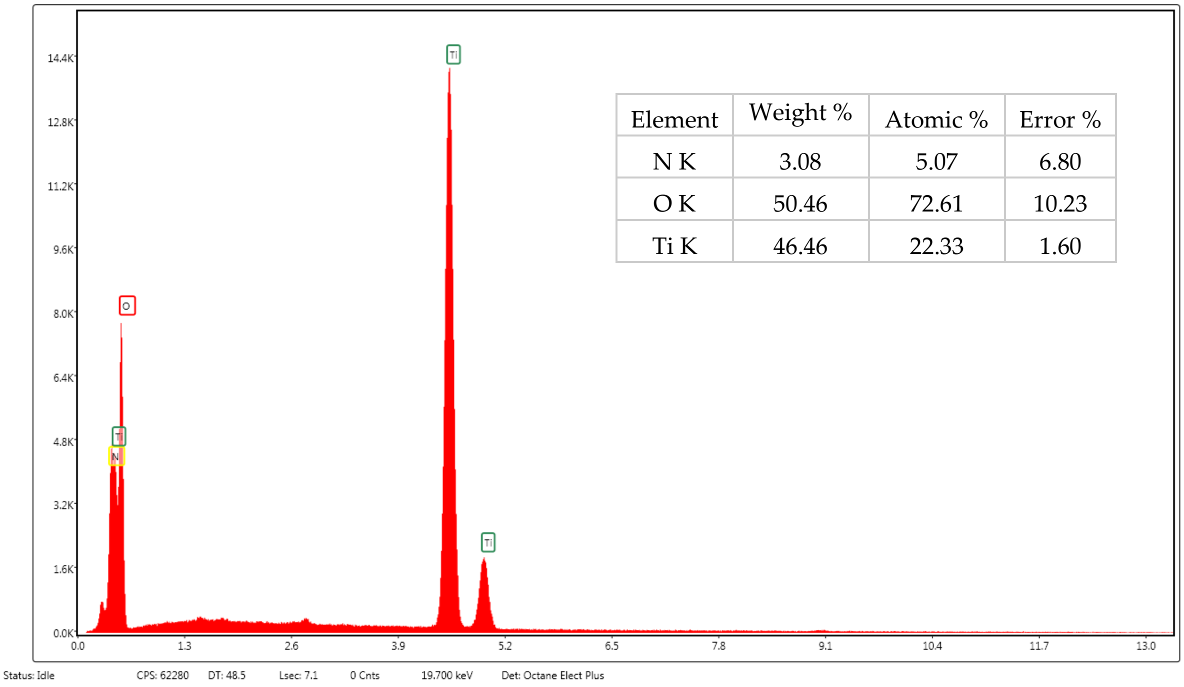

Element	Weight %	Atomic %	Error %
N K	3.08	5.07	6.80
O K	50.46	72.61	10.23
Ti K	46.46	22.33	1.60

Figure S3. e) SEM morphology and elemental composition of N-TiO_2 catalyst.

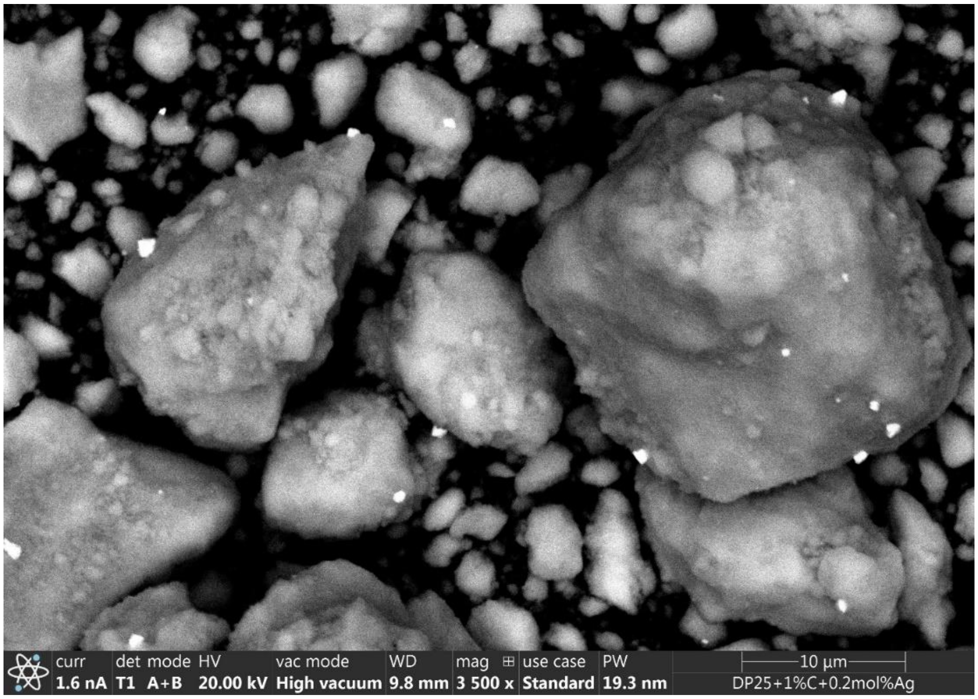

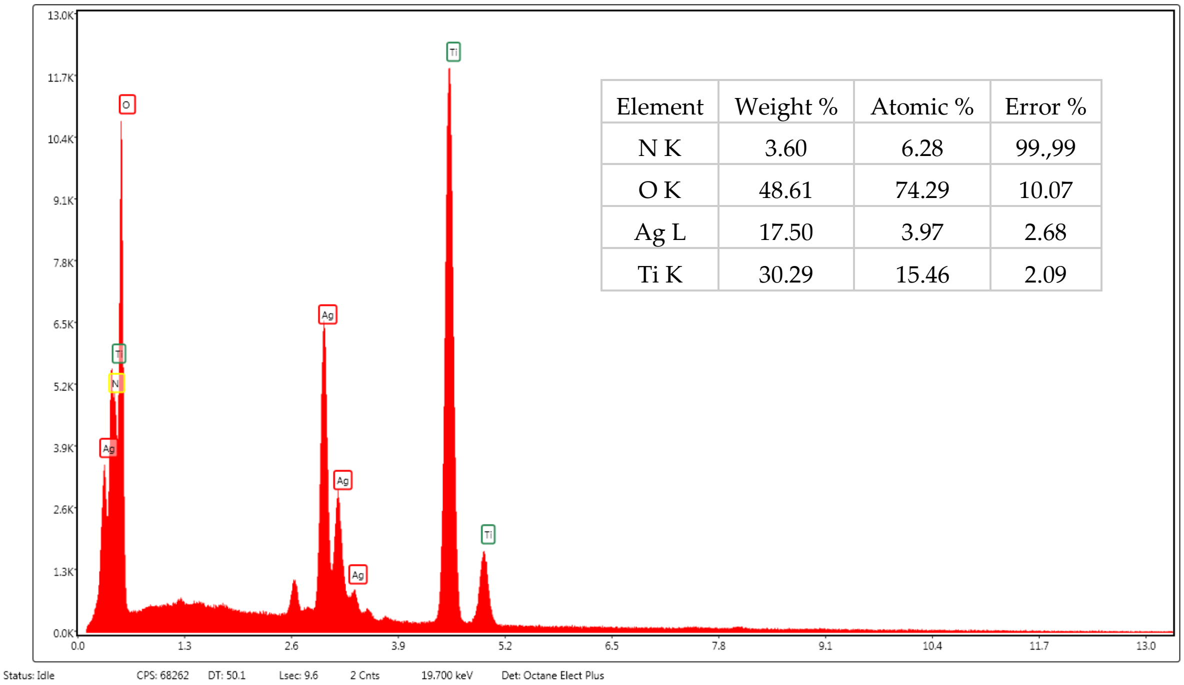

Element	Weight %	Atomic %	Error %
N K	3.60	6.28	99.,99
O K	48.61	74.29	10.07
Ag L	17.50	3.97	2.68
Ti K	30.29	15.46	2.09

Figure S3. f) SEM morphology and elemental composition of Ag-N-TiO_2 catalyst.

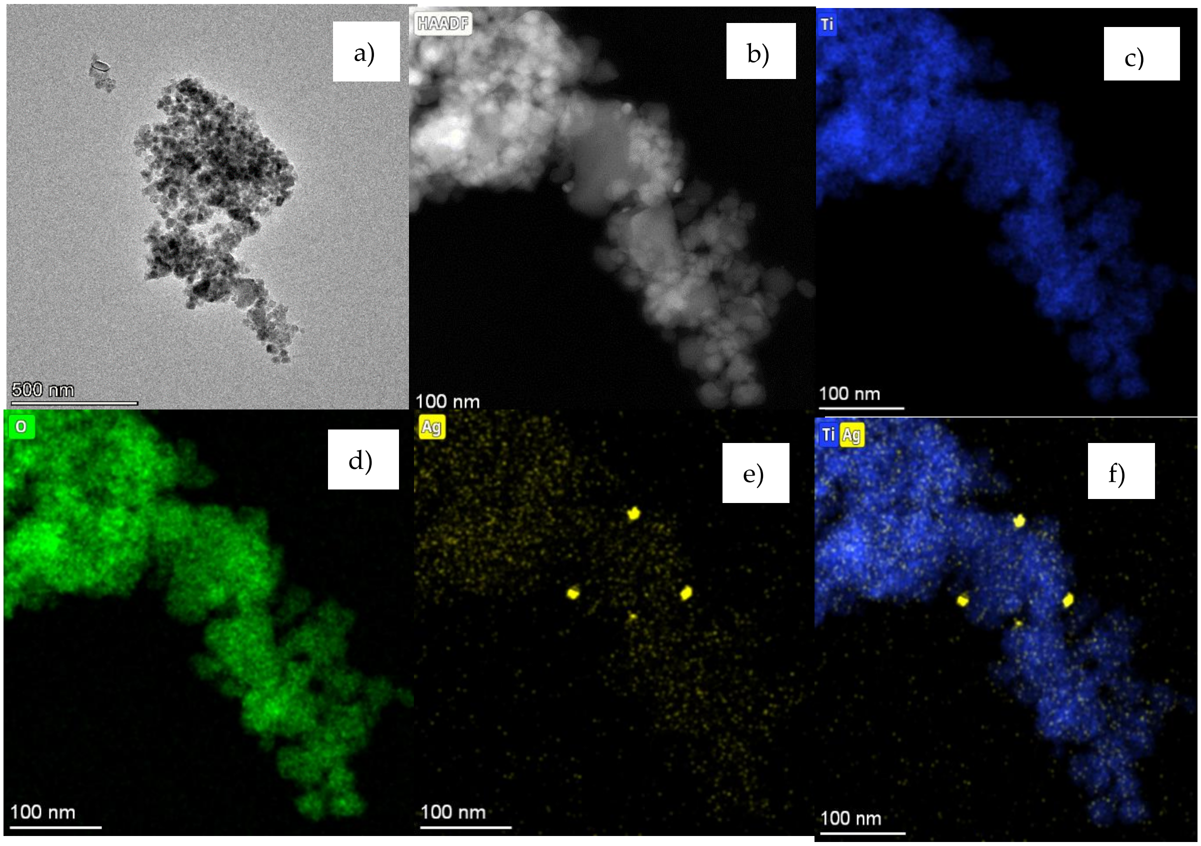

Figure S4. TEM analysis of the Ag-TiO_2 catalyst; (**a, b**) TEM micrographs with different magnifications; (**c-f**) element maps obtained in STEM–EDS mode.

Table S1. Band gaps of catalysts produced by mechanochemical treament (grinding).

Catalyst	g-DP25 TiO_2	N-TiO_2	Ag-TiO_2	N-Ag-TiO_2
**Band gap [eV]**	3.029	3.007	3.028	3.017
**Wavelength [nm]**	409	412	409	411
**Rutile content [%]**	24.4	16.6	25.8	21.2

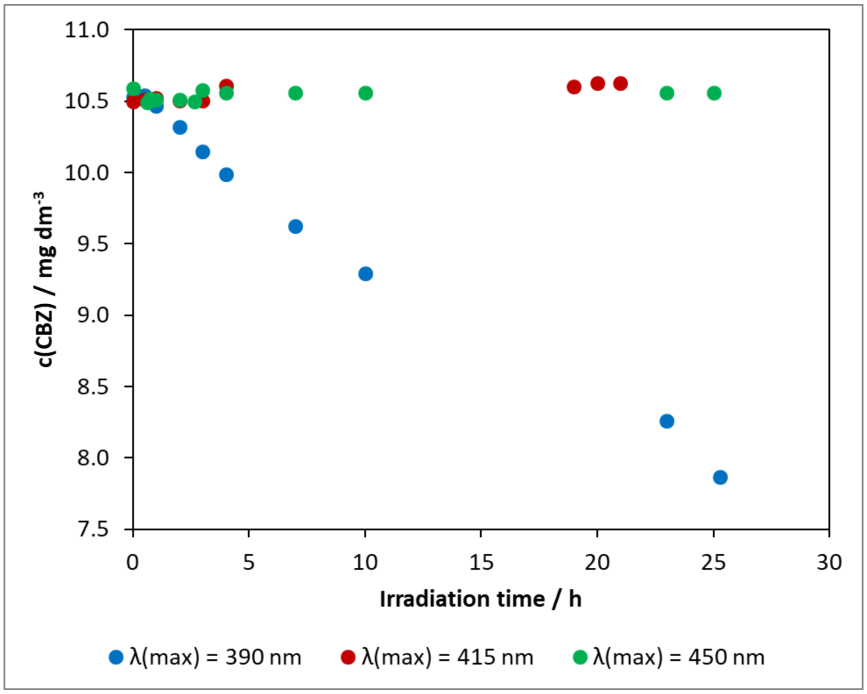

Figure S5. Changes in carbamazepine concentration during photolysis. $c_{(CBZ)0}$: 10 mg dm^{-3}, airstream: 10 dm^3 h^{-1}.

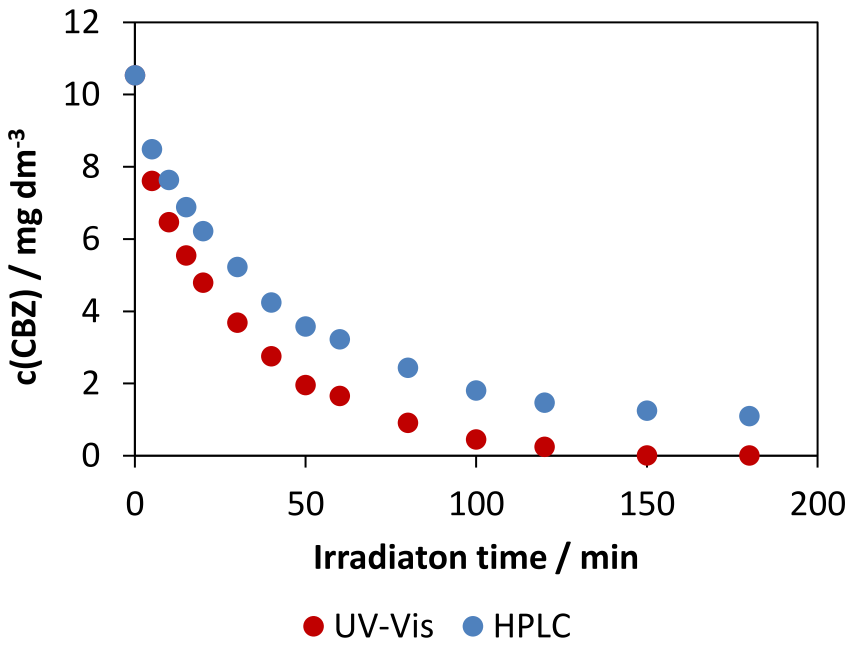

Figure S6. Changes in carbamazepine concentration, using DP25 TiO_2 photocatalyst. $c_{(CBZ)0}$: 10 mg dm^{-3}, airstream: 10 dm^3 h^{-1}, 1 g dm^{-3} DP25 TiO_2, l: 1 cm, $\lambda_{(det.)}$: 285 nm .

Table S2. Comparison of changes in COD measured during illuminations.

Catalyst	λ(max) = 390 nm			λ(max) = 415 nm			λ(max) = 450 nm		
	$COD_{t=0min}$	$COD_{t=180min}$	**ΔCOD**	$COD_{t=0\ min}$	$COD_{t=180min}$	**ΔCOD**	$COD_{t=0min}$	$COD_{t=180min}$	**ΔCOD**
DP TiO_2	24.67	0.80	**23.87**	25.17	17.50	**7.67**	24.78	20.23	**4.55**
g-DP TiO_2	27.25	13.50	**13.75**	23.50	21.20	**2.30**	25.68	22.58	**3.10**
N-TiO_2	25.11	21.50	**3.61**	25.56	22.00	**3.56**	24.12	22.87	**1.25**
Ag-TiO_2	23.25	19.30	**3.95**	24.71	18.50	**6.21**	24.58	22.36	**2.22**
N-Ag-TiO_2	24.22	19.89	**4.33**	24.44	22.67	**1.77**	25.24	22.54	**2.70**

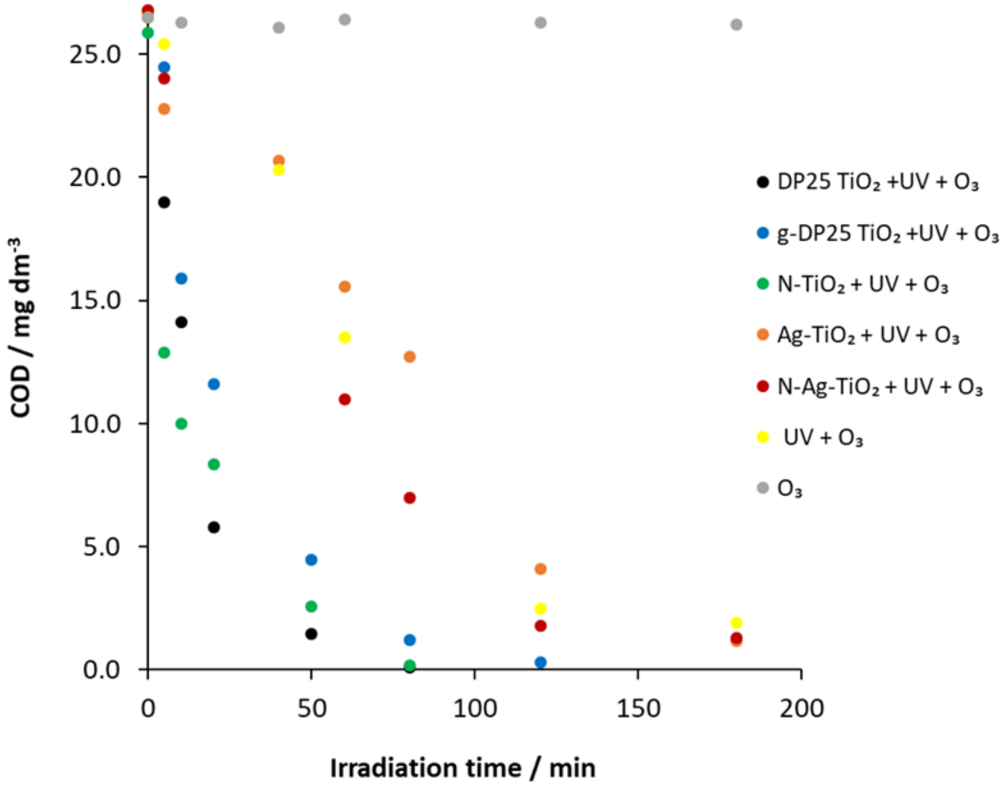

Figure S7. Changes in COD in carbamazepine solution during various treatments.

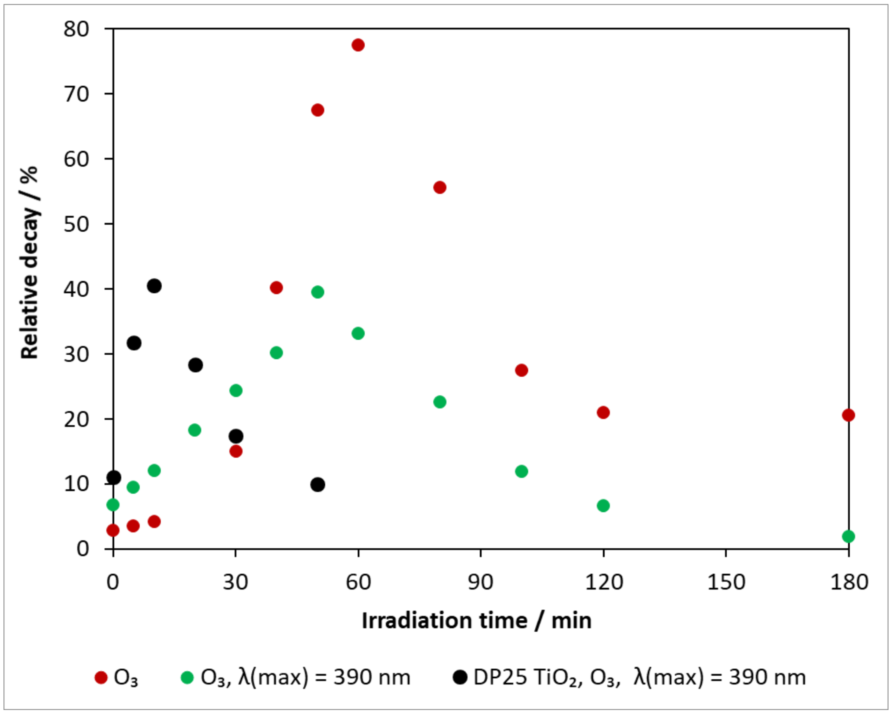

Figure S8. Changes in toxicity in carbamazepine solution, using ozone in various combinations.

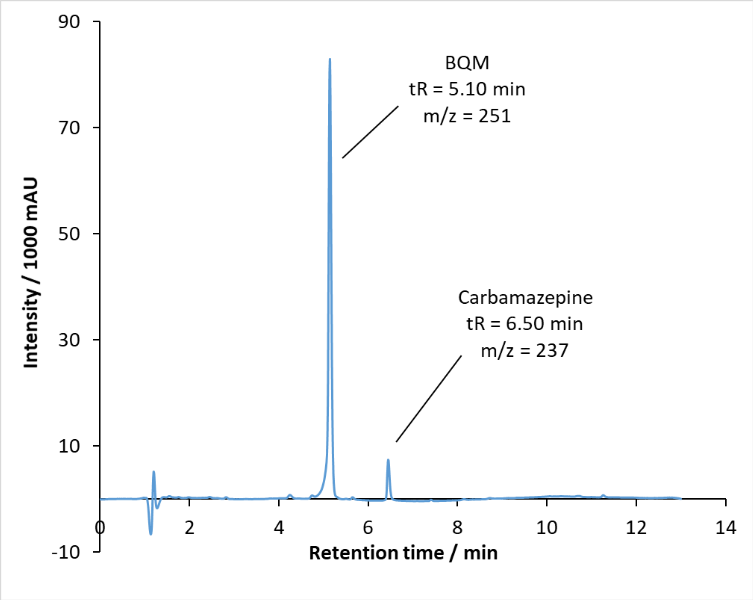

Figure S9. Chromatogram for the degradation of carbamazepine with ozone at 60 min reaction time.

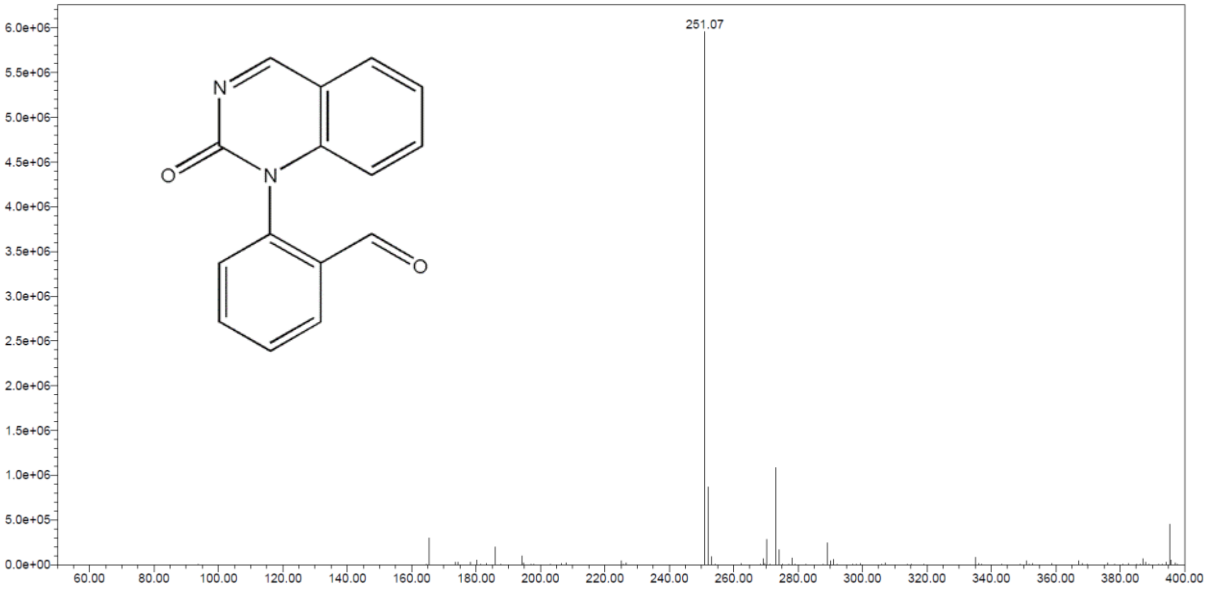

Figure S10. a) Mass spectrum of the compound with tR=5.1 min in the sample of CBZ ozonized for 60 min, and the structural formula of BQM (251 m/z).

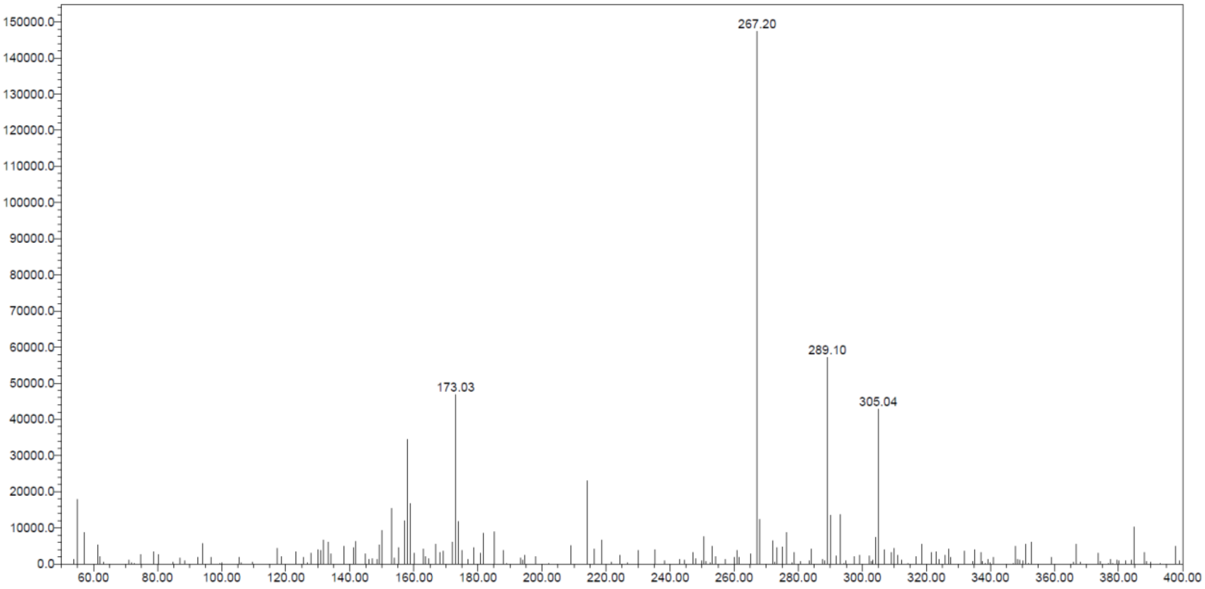

Figure S10. b) Mass spectrum of the compound (m/z 267) with tR=4.2 min in the sample of CBZ ozonized and irradiated for 50 min.

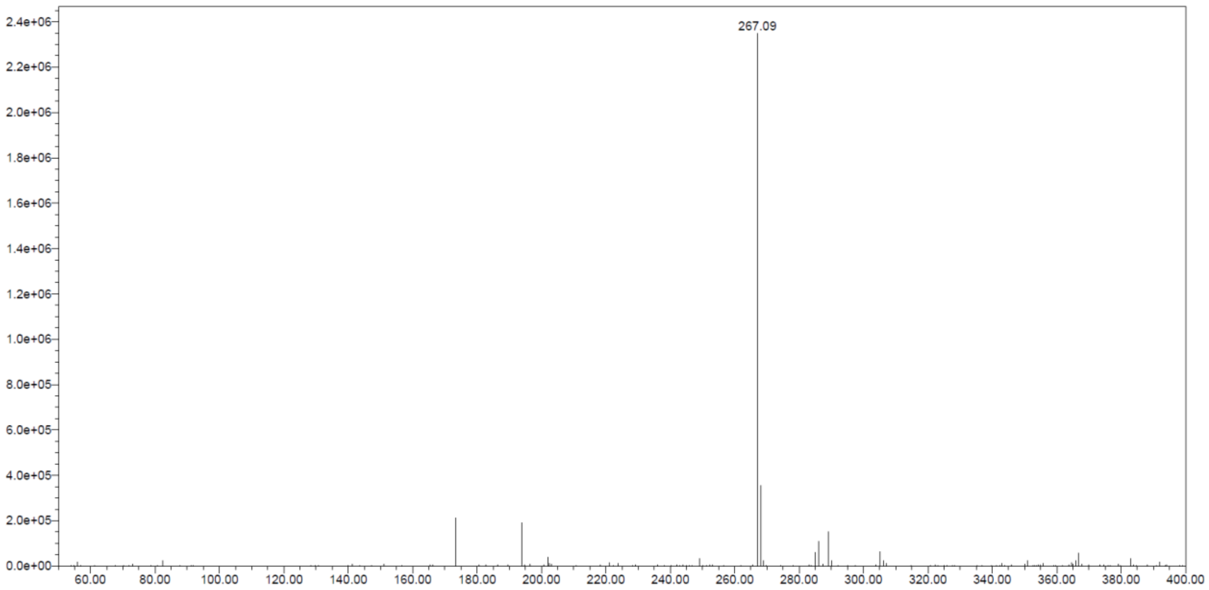

Figure S10. c) Mass spectrum of the compound (m/z 267) with tR=4.7 min in the sample of CBZ ozonized and irradiated for 50 min.

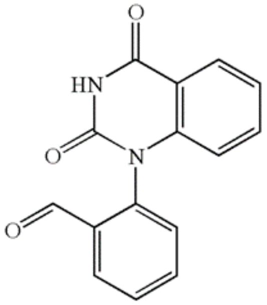

(a)

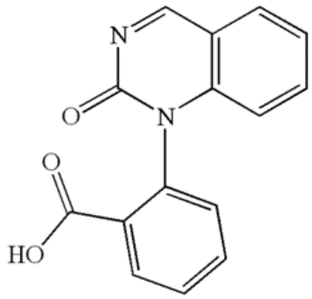

(b)

Figure S10. d) Structural formula of **(a)** BQD (m/z 267) and **(b)** BaQM (m/z 267).

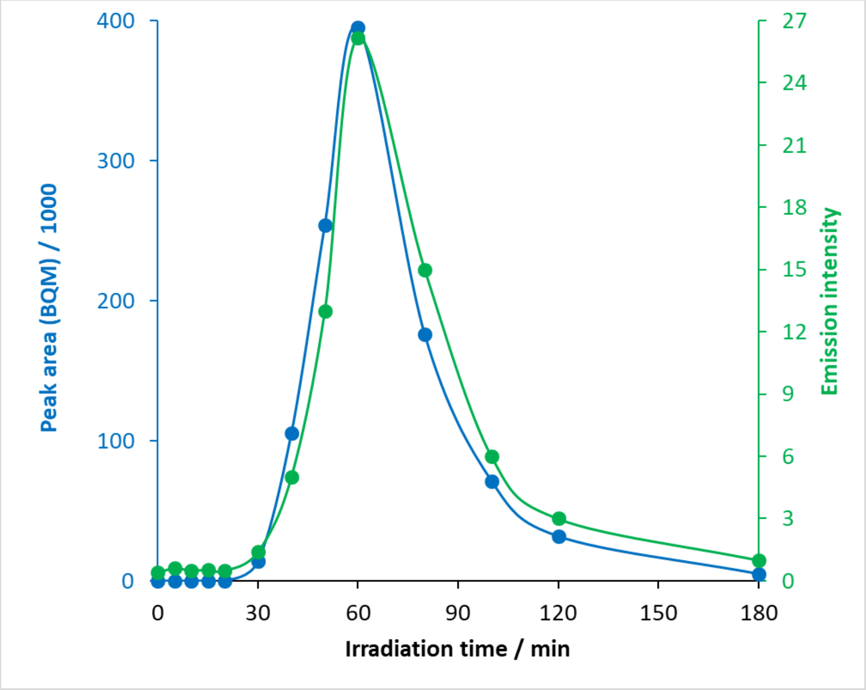

Figure S11. Peak areas and emission intensities of BQM during ozonization of carbamazepine.

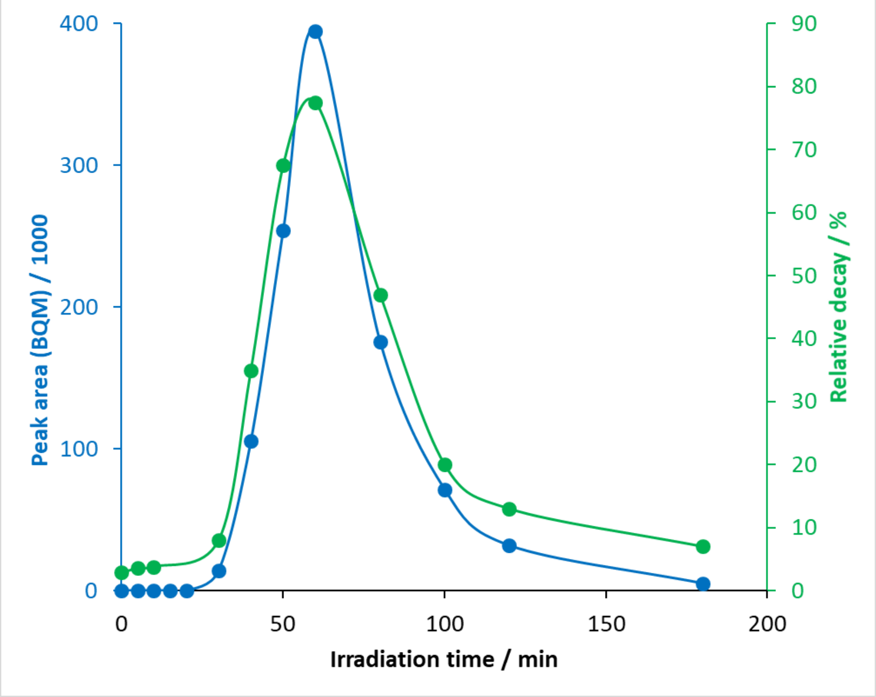

Figure S12. Peak areas of BQM and change in toxicity during ozonization of carbamazepine.

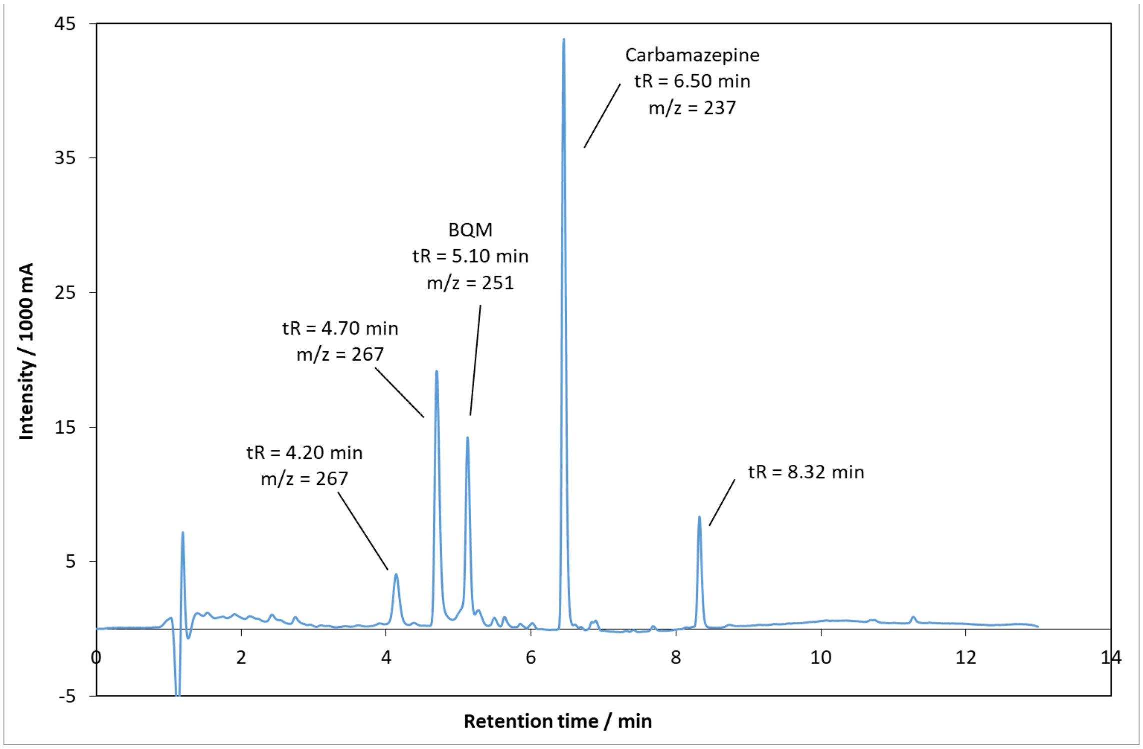

Figure S13. Chromatogram for the degradation of carbamazepine with ozone and UV light at 50 min reaction time.

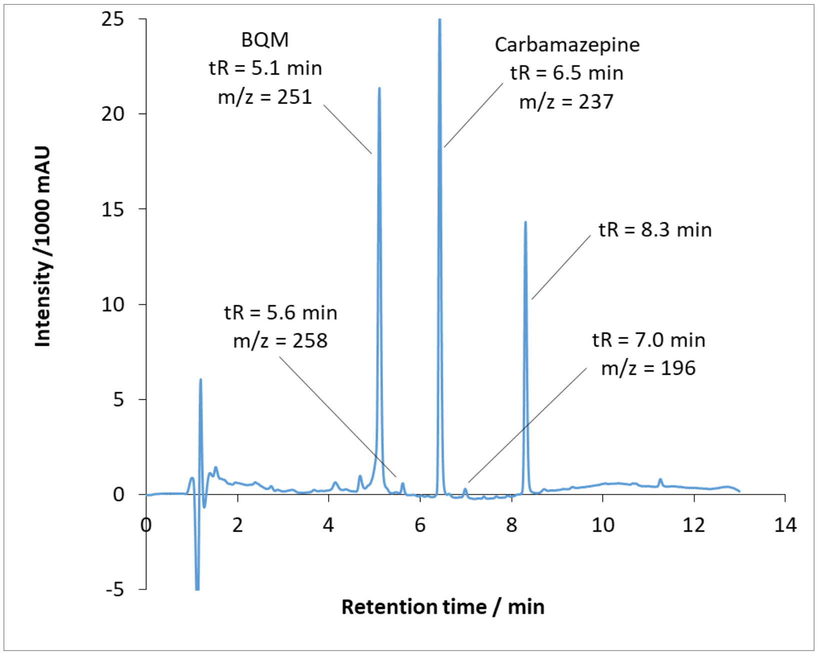

Figure S14. Fifteen min chromatogram for the degradation of carbamapezine, using N-TiO_2 + O_3 + UV.

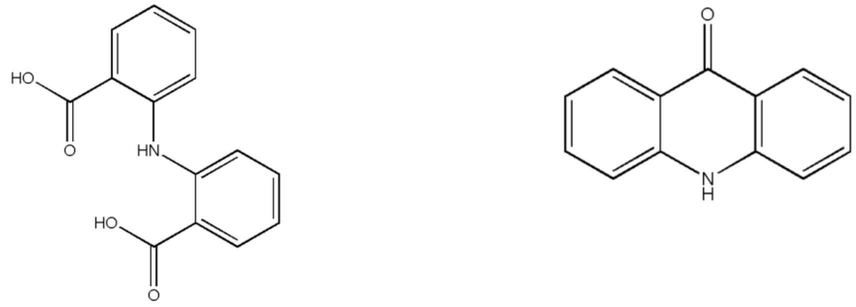

(**a**) (**b**)

Figure S15. Structural formula of **(a)** 2,2'-imino-dibenzoic acid (m/z 258) and **(b)** acridone (m/z 196).

Table S3. Intermediates formed under irradiation at various wavelength in the presence of DP25 catalyst.

tR [min]	λ(max) = 390 nm	λ(max) = 415 nm	λ(max) = 450 nm
4.70	✓	✗	✗
5.10	✓	✗	✓
5.45	✓	✓	✗
5.65	✓	✓	✓
6.00	✓	✓	✗
7.20	✓	✗	✗

✓ The intermediate product with the given retention time is formed.

✗ The intermediate with the given retention time is not formed.

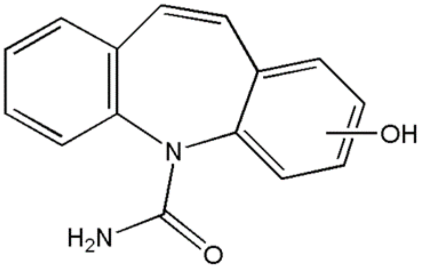

Figure S16. General structural formula of hydroxy-carbamazepine (m/z 253).

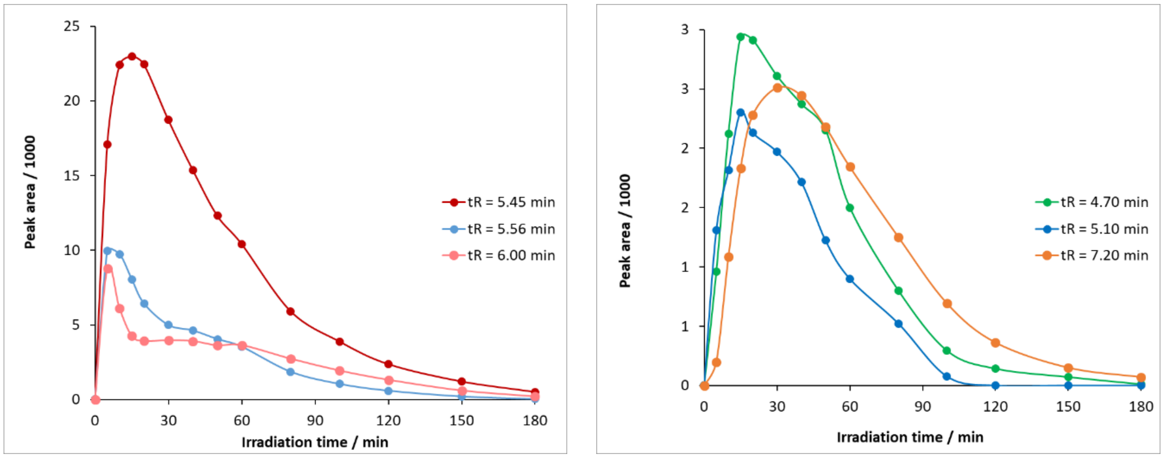

a) (b)

Figure S17. Temporal changes in the relative concentrations of intermediates of retention times a) 5.45, 5.56, and 6.00 min and b) 4.71, 5.10, and 7.20 min upon UV irradiation of carbamazepine solution on DP25 TiO_2 catalyst.

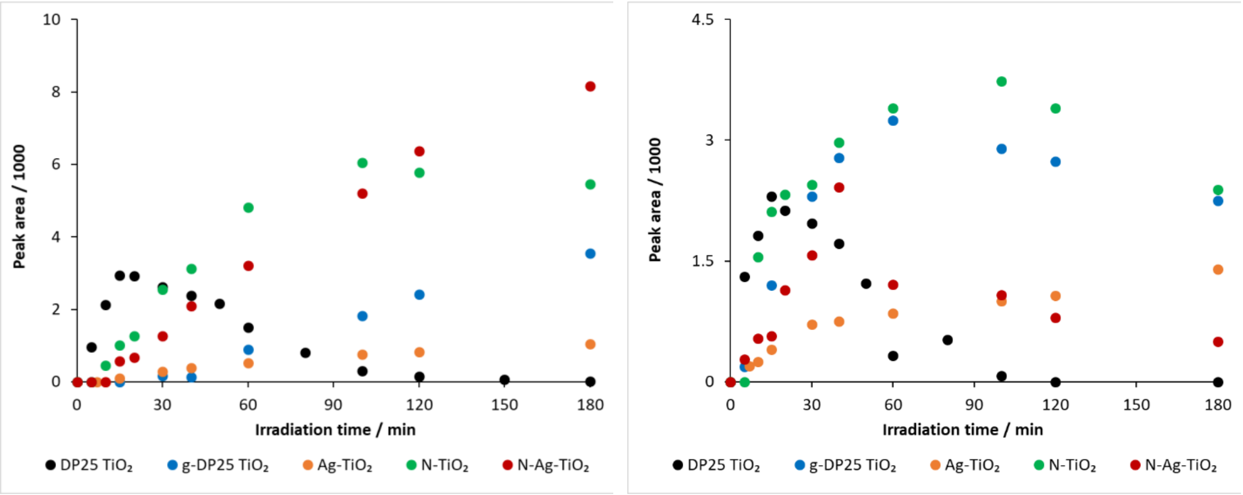

(a) (b)

Figure S18. Temporal changes in the relative concentrations of intermediates of retention time a) 4.70 min and b) 5.10 min upon UV irradiation of carbamazepine solution on various catalysts.

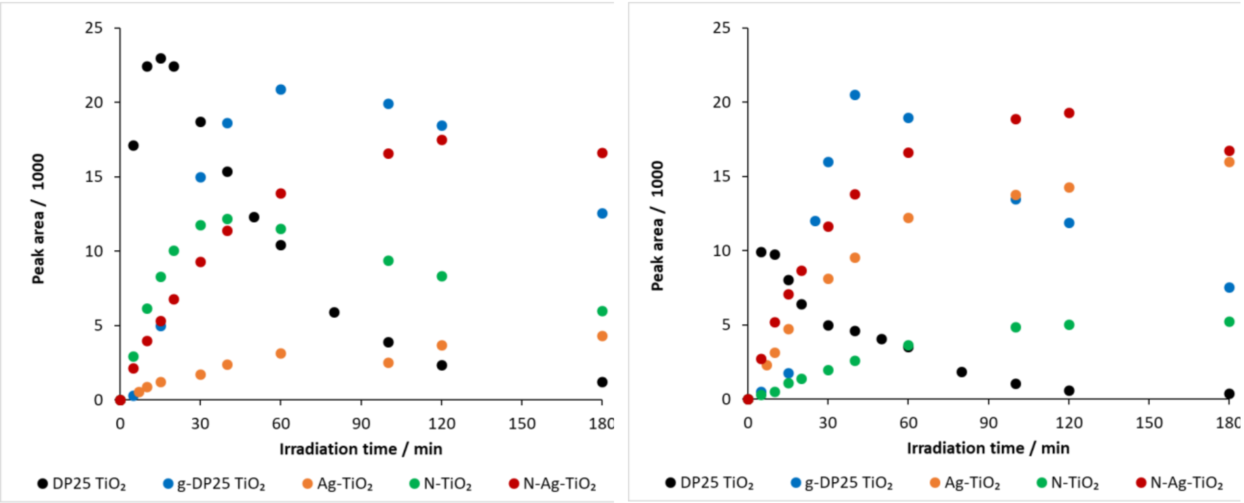

(**a**) (**b**)

Figure S19. Temporal changes in the relative concentrations of intermediates of retention time a) 5.45 min and b) 5.75 min upon UV irradiation of carbamazepine solution on various catalysts.

Table S4. Intermediates formed under irradiation at λ(max) = 450 nm in the presence of various catalysts.

tR [min]	DP25 TiO_2	g-DP25 TiO_2	N-TiO_2	Ag-TiO_2	N-Ag-TiO_2
4.57	✗	✓	✓	✗	✓
4.70	✗	✓	✓	✗	✓
5.10	✗	✓	✓	✗	✓
5.45	✓	✓	✓	✓	✓
5.65	✓	✗	✓	✓	✗

✓ The intermediate product with the given retention time is formed.

✗ The intermediate with the given retention time is not formed.

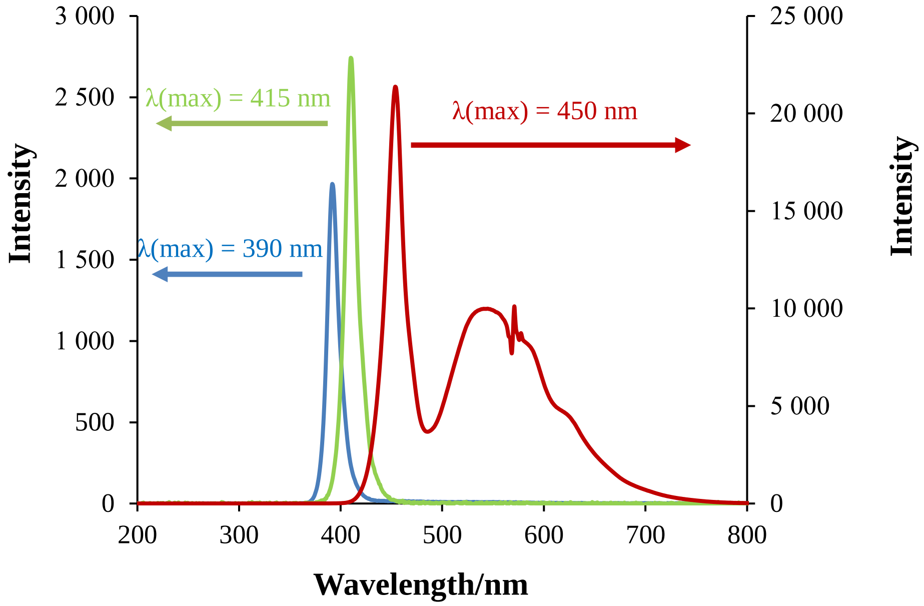

Figure S20. Emission spectra of the light sources applied.

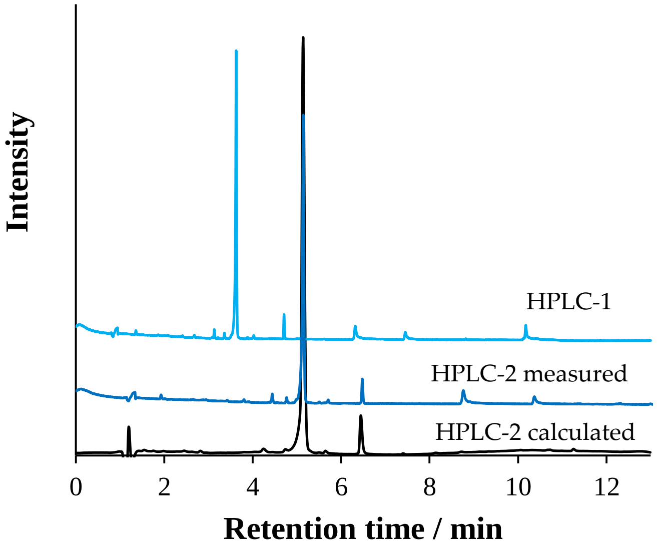

Figure S21. Relating the results of two HPLC systems to each other.

Text S1. Sample preparation for toxicity study

For measurements of the antibacterial effect, the catalyst samples were immobilized in an acrylate based polymer on a plastic surface. The catalyst concentration on the plastic surface was 485 mg/m^2. Discs of 1 cm diameter were cut from this plastic sheet and fixed to the bottom of small, open sample containers (glass tubes) with screw caps. The bacterial suspensions were layered on the samples. The toxicity effects of the catalysts were measured by using *Vibrio fischeri* luminescent bacteria. After reconstitution, the guaranteed life-span of bacteria was 4 h. The suspension of bacteria was incubated for 80 min at 15°C. At the end of the incubation period, 0.5 cm^3 sample was measured into the sample holders, which were kept in the dark during the experiment, and the emission intensities of the samples were recorded at defined times. The reference sample was stored in a bottle, which did not contain any plastic disc. The control sample contained a disc from a commercially available plastic sheet with antibacterial surface. During the evaluation, the results obtained from 3 parallel measurements were averaged and then the relative decomposition percentage was calculated. Afterwards, the relative decomposition (%) of the actual sample was compared to the control one.
